# *De novo* Genome Assembly, Annotation, and Comparative Analysis of the Lined Sole *Achirus lineatus* as a Resource for Evolutionary and Environmental Genomics

**DOI:** 10.1007/s10126-026-10665-8

**Published:** 2026-06-30

**Authors:** Mercedes Quintanilla-Mena, Elsa B. Góngora-Castillo, Rossanna Rodriguez-Canul, Rafael Rivera-Bustamante

**Affiliations:** 1 Departamento de Recursos del Mar, Cinvestav- Unidad Mérida, Km 6 Carretera Antigua a Progreso, Mérida, 97310 Yucatán México; 2 Secihti-Departamento de Recursos del Mar, Cinvestav Unidad Mérida, Km 6 Carretera Antigua a Progreso, Mérida, 97310 Yucatán México

**Keywords:** *Achirus lineatus*, Mitogenome, Comparative genomics, Ecotoxicology

## Abstract

The lined sole, *Achirus lineatus*, is a widely distributed species in the Gulf of Mexico. This basin is constantly exposed to hydrocarbon contamination due to natural oil seeps, oil extraction and oil spills. Previous studies suggest the line sole *A. lineatus* as a possible sentinel species for oil spills; but the overall landscape of xenobiotic metabolism in this species remains poorly understood. Access to high-quality reference genomes will improve this situation. Here, we report the first whole-genome sequence, assembly, and annotation of the lined sole *A. lineatus*. We generated 132 Gb of PacBio High-Fidelity (HiFi) reads, which were filtered to retain 93 Gb of ultra-high-quality data. The assembly resulted in a mitogenome of 16,579 bp and a nuclear genome of 486.3 Mb distributed across 199 contigs, with a contig N50 of 9.87 Mb. Benchmarking Universal Single-Copy Orthologs (BUSCO) analysis based on the *actinopterygii*_odb10 database (3,640 orthologs) identified 3,611 (99.2%) complete BUSCOs. Genome annotation strategy resulted in the identification of 22,412 protein-coding genes with a high percentage (95.94%) of functional annotation. The genome of *A. lineatus* revealed 693 expanded and 3349 contracted gene families. We also identified 123 genes involved in the xenobiotics biodegradation and metabolism pathways, as well as neuroplasticity-related genes that may be associated with responses to environmental toxicants, providing baseline genomic information that suggests an integrated adaptive strategy involving both metabolic and nervous system processes. This assembly represents a valuable genomic resource for future toxicological and ecological studies of this species.

**Supplementary Information:**

The online version contains supplementary material available at 10.1007/s10126-026-10665-8.

## Introduction

**Fig. 1:**
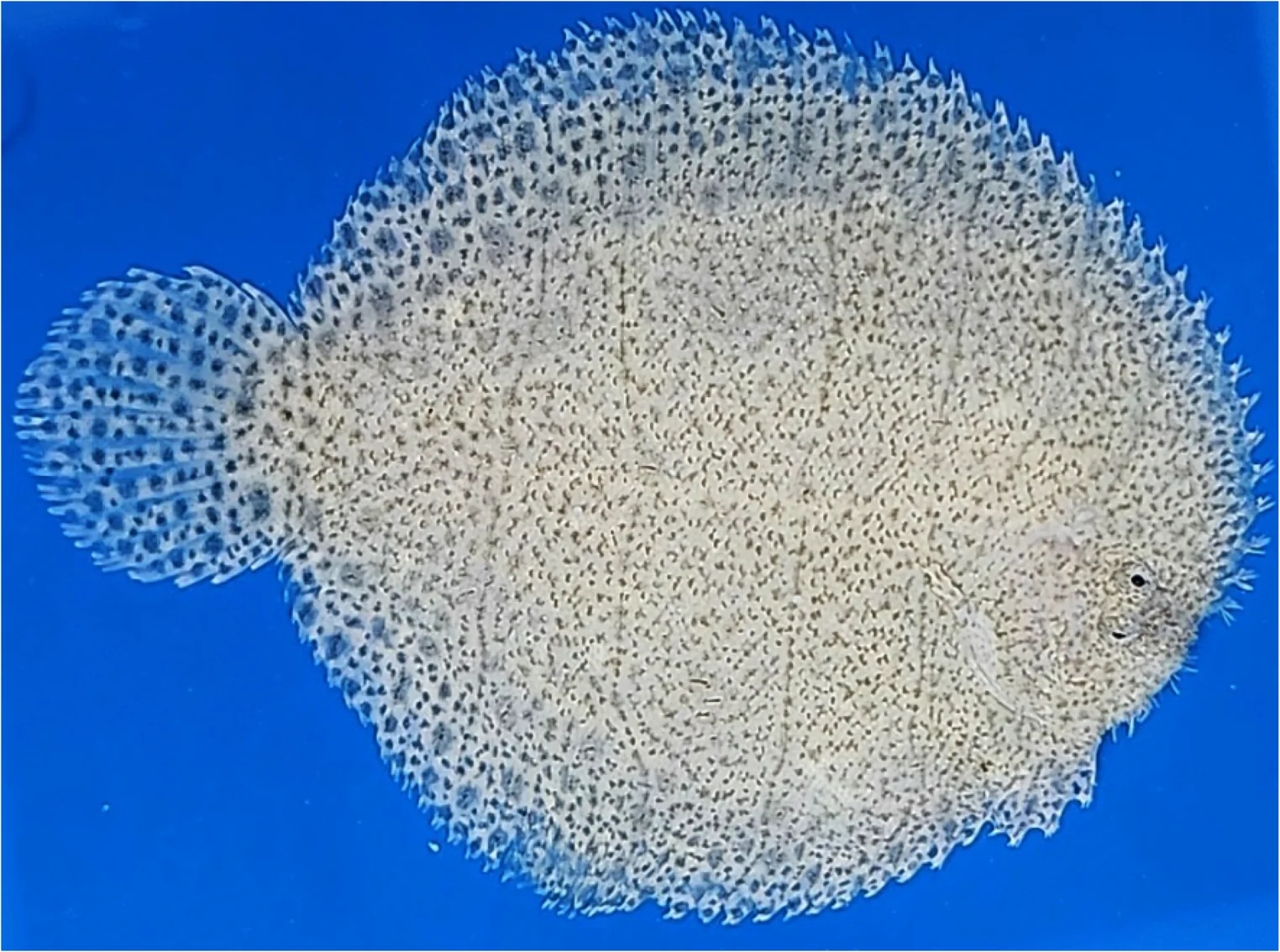
*Achirus lineatus* (lined sole) collected in the costal lagoon of Celestún, Yucatán, Mexico (20°49′40.00″ N, 90°25′22.88″ W). Original photo taken for this study

The lined sole, *Achirus lineatus*, is a small (23 cm maximum length), oval-shaped estuarine flatfish from the family *Achiridae*, with both eyes located on the right side of the body (de Oliveira and Fávaro 2010; Gracian Negrete 2012) (Fig. 1). It is a gregarious and benthic species that remains in permanent contact with marine sediments (Amendola-Pimenta et al. 2020). It is widely distributed across the coastal Atlantic lagoons of the American continent, ranging from Florida to Uruguay (Robins et al. 1986; de Oliveira and Fávaro 2010). It is a common species from all coastal lagoons of the Gulf of Mexico (GoM) throughout the year. Adults are more frequently observed in summer and juveniles in fall and winter seasons (Kobelkowsky 2000; de Oliveira and Fávaro 2010; Rocha et al. 2014). *A. lineatus* tolerates broad salinity gradients, as coastal lagoons in the GoM experience pronounced salinity variability (∼5–48 psu) (Chuang et al. 2017; Hardage et al. 2022), which contributes to the ecological versatility of this flatfish species.

However, this species has been included in the historical exposure to hydrocarbon contamination because some areas of the Gulf of Mexico have suffered for thousands of years due to natural oil seepage. Furthermore, during the last century, intensive oil extraction in the Gulf of Mexico, including major spills such as Ixtoc I (1979), Taylor Energy (2004), and Deepwater Horizon (2010) (Pulster et al. 2020) have increased its exposure to hydrocarbon pollution. In the most recent spill event (February 2026), a pipeline leak at PEMEX (Petróleos Mexicanos) facilities was reported to affect around 630 km of coastal ecosystems from Campeche to Tamaulipas (PEMEX 2026). This situation has raised concern for the populations of marine organisms inhabiting this basin, leading to environmental monitoring efforts within the GoM to assess hydrocarbon contamination as well as the responses of molecular biomarkers in other flatfish from the same area (Puch-Hau et al. 2016; Quintanilla-Mena et al. 2020; Cañizares-Martínez et al. 2024b).

Significant concentrations of hydrocarbons have been detected in marine sediments from coastal lagoons and offshore waters of the GoM (Quintanilla-Mena et al. 2020). To facilitate the monitoring of these hydrocarbons and their effect on present fauna, a small number of biomarkers have been established. The biomarkers are related to the biotransformation pathways, which modify the structure of xenobiotics into more hydrophilic molecules (phase I enzymes), facilitating their elimination from the body (phase II enzymes) as well as with stress response pathways (e.g., antioxidant proteins) in fishes (Zhu et al. 2016; Quintanilla-Mena et al. 2020; Cañizares-Martínez et al. 2021, 2024a; Franco et al. 2025). However, a deeper understanding of the mechanisms that enable these species to tolerate contaminated areas is highly required.

In previous toxicological bioassays with hydrocarbons in *A. lineatus*, this organism was suggested as a possible sentinel species for oil spill events (Amendola-Pimenta et al. 2020; Cerqueda-García et al. 2020; Zamora-Briseño et al. 2021; Cañizares-Martínez et al. 2024a). However, the overall landscape of xenobiotic metabolism in *A. lineatus* remains poorly understood, because working with non-model species poses additional methodological challenges that limit studies of physiological and genetic adaptation, molecular regulation, and evolutionary processes.

Access to high-quality reference genomes will improve our ability to uncover genetic mechanisms and evolutionary dynamics in stress-resistant species (Johnston et al. 2025). Thus, obtaining a high-quality reference genome for *A. lineatus* represents a key step in the process of understanding the molecular basis of contaminant tolerance and supports its possible role as a sentinel species in environmental monitoring programs.

In this study, we report the *de novo* genome sequencing, assembly and annotation, including the mitogenome of *A. lineatus* from Celestún, Yucatán [a coastal lagoon in the southern GoM (20°49′40.00″ N, 90°25′22.88″ W)]. We further performed a comparative genomic analysis with other flatfish species to identify species-specific gene families and functional categories associated with possible contaminant responses.

## Materials and methods

### Sample Collection and DNA Extraction

On May 11, 2024, several specimens of *A. lineatus* (average total length = 8 cm) were captured in the coastal lagoon of Celestún, Yucatán, Mexico (20°51′30″N 90°24′0″W), using an artisanal riverine fishing gear known as a triangular shrimp net (0.5 cm mesh size). All live animal protocols described below were approved by institutional ethic committee (Comité Institucional de Cuidado y Uso de Animales of the Centro de Investigación y de Estudios Avanzados, CICUAL-Cinvestav), under the reference number 0419 − 051. The procedures followed the guidelines established in the Mexican Official Standard NOM-062-ZOO-1999.

Several extraction protocols were performed using tissues such as liver, intestine, gills, eyes, and brain. The highest DNA quality and integrity were obtained from liver and gill samples. Therefore, these tissues were selected to achieve the optimal DNA quality and quantity required for sequencing. One of the juvenile specimens(total length = 7.9 cm; body weight = 5.6 g), with the best DNA integrity and concentration was selected for the genomic analysis. Liver and gill tissues were dissected and frozen in liquid nitrogen during transportation, then stored at − 80 °C in the laboratory until further processing. For genomic DNA extraction ~ 25 mg of each tissue was macerated in liquid nitrogen and DNA was extracted using the DNeasy Blood & Tissue Kit (QIAGEN) following the manufacturer’s protocol. The extracted DNA was treated with 0.2 µL of RNase A (10 mg/mL) (SIGMA-ALDRICH) for 30 min at 37 °C. DNA samples were evaluated using NanoDrop 2000 spectrophotometer (Thermo Fisher Scientific, USA), agarose gel electrophoresis, and Qubit fluorometer (Invitrogen, USA). DNA from both tissues was pooled for sequencing.

### Library Preparation and PacBio Long-Read Sequencing

A PacBio HiFi library was constructed using the SMRTbell library preparation protocol (library concentration = 34.2 ng/µL; average fragment size = 13,627 bp) and sequenced on a PacBio Revio system by Macrogen (Seoul, South Korea).

### Sequence Quality Verification, Filtering and Genome Size Estimation

Quality and size distribution of 132 Gb of raw data was evaluated using NanoPlot v1.46.1 (De Coster and Rademakers 2023). Sequencing adapters were verified using HiFiAdapterFilt v1.0.0 (https://github.com/sheinasim-USDA/HiFiAdapterFilt). Ultra–high-quality HiFi reads (QV > 30 and read length > 10,000 bp) were retained with chopper v0.8.0 (De Coster and Rademakers 2023). K-mer frequency analysis (k = 21) was performed with Meryl v1.4 (Rhie et al. 2020), and the k-mer–based genomic profile (genome size, heterozygosity, and repeat content) were estimated using GenomeScope v2.0 (Ranallo-Benavidez et al. 2020).

### Genome Assembly and Quality Control of the Assembly

After filtering, the PacBio clean HiFi reads were used to generate a contig-level genome assembly using Hifiasm v0.19.9-r616 using the parameters -t 16 -D 10 (Cheng et al. 2021). The assembly was then analyzed with BlobTools v1.1.1 (Laetsch and Blaxter 2017) to verify cross-contamination using the NCBI nucleotide collection database (nt v1.1 2024). The final assembly was analyzed using QUAST v5.2.0 (Mikheenko et al. 2023) and Benchmarking Universal Single-Copy Orthologs (BUSCO v5.7.0) (Simão et al. 2015; Manni et al. 2021a) with default parameters and the *actinopterygii_odb10* database. The assembly accuracy and k-mer completeness were assessed with Merqury v1.3 (Rhie et al. 2020), and read mapping–based quality assessment was performed with pbmm2 v1.14.99 (https://github.com/PacificBiosciences/pbmm2).

### Mitogenome Assembly

The mitochondrial genome was *de novo* assembled and annotated using the MitoHiFi (3.2.3 + galaxy0) pipeline (Uliano-Silva et al. 2023), with default parameters on the Galaxy USA instance (Galaxy version 25.1.rc1), and subsequently visualized with MitoFish (2025.06) (Iwasaki et al. 2013; Sato et al. 2018; Zhu et al. 2023).

### Repeat Content Analysis

A *de novo* repeat library was constructed with RepeatModeler v2.0.5 (Flynn et al. 2020) using RepeatScout v1.0.6 (Price et al. 2005) and RECON v1.08 (Bao and Eddy 2002) with default parameters. Long Terminal Repeat (LTR) structural analysis was performed with LTR_harvest v1.6.2 (Ellinghaus et al. 2008) followed by refinement with LTR_retriever v2.9.0 (Ou and Jiang 2018) under RepeatModeler v2.0.5 pipeline (parameter: -LTRStruct). Homology-based detection was carried out using the *de novo* repeat library generated in the previous step to identify known repeat elements with RepeatMasker v4.1.7-p (Smit et al. 2015) and Dfam v3.8 database, which integrates TandemRepeatsFinder v4.09 (Benson 1999) in its pipeline. Finally, RepeatMasker was used to soft-mask simple repetitive regions and hard-mask complex regions. The resulting masked genome was employed for structural annotation.

### Gene Prediction and Functional Annotation

MAKER v2.31.11 (Cantarel et al. 2008) pipeline was used for genome annotation. Ab initio prediction was performed using the gene predictors AUGUSTUS v3.5.0 (Stanke et al. 2008) and SNAP v2.68.5 (Korf 2004). In parallel, homology-based prediction relied on protein sequences from *Danio rerio* (RefSeq assembly accession: GCF_000002035.6) and nine additional flatfish species, including *Cynoglossus semilaevis* (RefSeq assembly accession: GCF_000523025.1; UniProt proteome: UP000265120), *Hippoglossus hippoglossus* (RefSeq assembly accession: GCF_009819705.1), *Hippoglossus stenolepis* (RefSeq assembly accession: GCF_022539355.2), *Paralichthys olivaceus* (RefSeq assembly accession: GCF_024713975.1), *Platichthys flesus* (RefSeq assembly accession: GCF_949316205.1), *Pleuronectes platessa* (UniProt proteome: UP001153269), *Scophthalmus maximus* (RefSeq assembly accession: GCF_013347765.1; UniProt proteome: UP000246464), *Solea senegalensis* (UniProt proteome: UP000693946), and *Solea solea* (GenBank assembly accession: GCA_958295425.1). Protein sequences were retrieved from NCBI, Ensembl and UniProt databases, and initially aligned to the *A. lineatus* genome using TBLASTn tool v2.9.0 (Camacho et al. 2009) (*E*-value ≤ 1*e*^− 5^). Refined protein-to-genome alignments were subsequently generated using Exonerate v2.4.0 with the protein2genome model within the MAKER pipeline.

For the transcriptome evidence, RNA-seq data from the liver and gills of *A. lineatus* exposed and unexposed to the water-accommodated fraction (WAF) of oil (BioProject: PRJNA646280) (Zamora-Briseño et al. 2021) were downloaded and *de novo* assembled with Trinity v2.15.1(Grabherr et al. 2011) to generate a reference transcriptome. Read-mapping coverage was assessed by aligning the raw reads to the assembled transcriptome using Bowtie2 v2.4.5 (Langmead and Salzberg 2012), and transcriptome completeness was evaluated with BUSCO. Transcriptomic evidence was aligned to the genome using Exonerate with the est2genome model as implemented in MAKER, providing exon–intron structure support for gene prediction. The three gene model predictions were merged using MAKER in three iterative rounds to refine the structural annotation. To refine transcript boundaries, splice junctions, and structural gene models, PASA v2.5.3 (Haas et al. 2003, 2008) was integrated into the annotation pipeline (MAKER+PASA) using the same transcriptomic evidence employed during the MAKER annotation process. Transcript alignments to the genome assembly were generated using GMAP and BLAT, and PASA was subsequently used to update existing MAKER gene models.

To assess the quality of the predicted proteome obtained, protein completeness was evaluated using BUSCO (Simão et al. 2015; Manni et al. 2021b) based on single-copy orthologs, and Orthology-based Markers for Annotation Quality (OMArk v0.3.1) using conserved Hierarchical Orthologous Groups (HOGs). In addition, OMArk was applied to the entire proteome to estimate the proportion of accurately predicted versus erroneous gene models and to detect potential contamination from non-target species (Nevers et al. 2025).

Functional annotation of predicted protein-coding genes was conducted using BLASTp v2.9.0+ (*E*-value ≤ 1*e*^− 5^) against UniProt-UniRef90 release 2025 (Suzek et al. 2014); BlastKOALA v3.1 (Kanehisa et al. 2016) for Kyoto Encyclopedia of Genes and Genomes KEGG-based functional annotation (release 115.0 2025); KofamKOALA release 115.0 2025 (Aramaki et al. 2020) employing HMMER v3.1 (Eddy 2011) for KEGG ortholog assignment; and eggNOG-mapper v2.1.12 using DIAMOND v2.1.11 (Buchfink et al. 2021) searches against the EggNOG database (eggnog.db v5.0.2), which contains providing orthology-based functional annotation including Gene Ontology (GO) terms, KEGG pathways, COG/KOG functional categories, Pfam domains, and evolutionary orthologous group assignments (Huerta-Cepas et al. 2019; Cantalapiedra et al. 2021; Hernández-Plaza et al. 2023).

### Reference-Guided Scaffolding

Reference-guided scaffolding was conducted using the chromosome-level genome of a phylogenetically related flatfish species within the same order (Pleuronectiformes) (Liu et al. 2018), the olive flounder *P. olivaceus* (RefSeq accession: GCF_024713975.1), which diverged approximately 49 million years ago (Kumar et al. 2022). Among the currently available high-quality and complete flatfish chromosome assemblies cataloged in GoaT (GenomeHubs Open Annotation Tool; version 2025.04.21; https://goat.genomehubs.org), *P. olivaceus* represents the closest phylogenetic relative to *A. lineatus*. For this reason, it was selected as the reference genome for scaffolding.

Scaffolding was performed using RagTag v2.1.0 (Alonge et al. 2022). To assess large-scale assembly after reference-guided scaffolding, the proportion of contigs aligned to the *P. olivaceus* reference chromosomes was evaluated. Because the scaffolding process relied on the *P. olivaceus* reference genome, this comparison was intended solely as an internal validation of assembly contiguity, regardless of chromosome number or order, rather than as an assessment of structural conservation between species.

### Comparative Genome Analysis

Comparative genomics analysis was conducted to infer phylogenetic relationships and evolutionary patterns using OrthoFinder v3.1.0 (-M msa) (Emms and Kelly 2019) and IQ-TREE v1.6.12 (-st AA -m MFP -bb 1000 -alrt 1000 -bnni -safe -nt 30) (Nguyen et al. 2015; Thi Hoang et al. 2017; Kalyaanamoorthy et al. 2017). OrthoVenn3 (Sun et al. 2023) was used for comparative visualization of orthologous gene clusters based on OrthoFinder clustering, using an inflation parameter of 1.20.

The proteome of seven species were included in this analysis: *D. rerio* (RefSeq assembly accession: GCF_000002035.6), *C. semilaevis* (RefSeq assembly accession: GCF_000523025.1; UniProt proteome: UP000265120), *H. hippoglossus* (RefSeq assembly accession: GCF_009819705.1), *H. stenolepis* (RefSeq assembly accession: GCF_022539355.2), *P. olivaceus* (RefSeq assembly accession: GCF_024713975.1), *P. flesus* (RefSeq assembly accession: GCF_949316205.1), and *S. solea* (GenBank assembly accession: GCA_958295425.1). Protein sequences were retrieved from the NCBI, Ensembl, and UniProt databases. The longest protein isoform per gene locus was selected using the OrthoFinder utility script primary_transcript.py for proteomes obtained from Ensembl and NCBI. No redundant reference proteome was retrieved for *C. semilaevis* from UniProt, and the predicted *A. lineatus* proteome contained a single representative protein sequence per gene model.

A maximum likelihood species phylogenetic tree was inferred using IQ-TREE from concatenated protein alignments of single-copy orthologs identified by OrthoFinder (-M msa). According to the Bayesian Information Criterion (BIC), the best-fitting substitution model under the maximum likelihood framework was Jones–Taylor–Thornton with empirical amino acid frequencies and a FreeRate model with four rate categories (JTT + F+R4). Branch support was assessed using the Shimodaira–Hasegawa approximate likelihood ratio test (SH-aLRT) and ultrafast bootstrap analyses.

Gene family expansions and contractions were subsequently analyzed using the final IQ-TREE phylogenetic tree as input for the Gene EXpansion and CONtraction analysis pipeline (EXCON v2.3.1) (Wyatt 2026), implemented using the nf-core framework (Ewels et al. 2020). EXCON performs ultrametric calibration of the phylogenetic tree while estimating the gene family gain/loss rate (λ) and automatically identifies the best-fit evolutionary model for ultrametric tree reconstruction. The resulting divergence-calibrated ultrametric tree was then used by CAFE v4.2.1(De Bie et al. 2006; Mendes et al. 2020) to infer gene family expansions and contractions.

Species-specific gene clusters and gene families were subjected to Gene Ontology (GO) enrichment analysis (Ashburner et al. 2000; Aleksander et al. 2026) using the OrthoVenn3 pipeline for species-specific genes (Sun et al. 2023) and the EXCON pipeline for expanded and contracted gene families. GO enrichment significance was evaluated using Fisher’s exact test implemented in TopGO, and multiple testing correction was performed.

To perform a comparative analysis of xenobiotic biodegradation and metabolism pathways, the proteomes of *A. lineatus*, *C. semilaevis*, *D. rerio*, *H. hippoglossus*, *H. stenolepis*, *P. olivaceus*, *P. flesus*, and *S. solea* were functionally annotated using eggNOG-mapper under the same methodological framework to obtain KEGG annotations. To ensure comparability among species, only the longest protein isoform per gene locus was retained for all proteomes prior to annotation, as described above. Genes assigned to xenobiotic biodegradation and metabolism pathways were subsequently classified into phase I and phase II biotransformation enzymes based on their annotated enzyme family names and functional descriptions. phase I enzymes included cytochrome P450s, monooxygenases, dehydrogenases, reductases, oxidases, dioxygenases, and hydrolases, whereas phase II enzymes comprised glutathione S-transferases (GSTs) and UDP-glucuronosyltransferases (UGTs). Genes that could not be assigned to either category were grouped as other xenobiotic-related enzymes.

To account for differences in proteome size and annotation completeness among species, gene counts were normalized as the number of genes assigned to each category per 1,000 genes with KEGG Orthology (KO) assignments. Differences in the relative abundance of xenobiotic metabolism gene categories between *A. lineatus* and the remaining flatfish species were evaluated using Fisher’s exact tests, followed by Benjamini–Hochberg false discovery rate (FDR) correction.

### *Cis*-Regulatory Element Analysis

*Cis*-regulatory elements (CREs) were analyzed for selected genes potentially involved in xenobiotic response. They were identified within *A. lineatus*-specific gene clusters obtained from the orthology analysis, to identify potential species-specific regulatory signatures associated with adaptation or response to environmental contaminants. Promoter regions (here considered the 2,000 bp upstream of the transcription start site) for these genes were extracted using TBtools-II v2.390 (Chen et al. 2023), and used for subsequent analyses. Motif scanning and CREs annotation were performed with FIMO v5.5.8 (Grant et al. 2011), as implemented in the MEME Suite v5.5.8 (Bailey et al. 2015), using the JASPAR 2024 CORE vertebrates non-redundant position frequency matrices. The resulting motif hits were manually curated to retain only transcription factors that were annotated in the *A. lineatus* genome. Finally, the CREs distributions within promoter regions were visualized using the BioSequence Viewer module of TBtools-II.

To statistically evaluate cis-regulatory element (CRE) enrichment in the promoter regions of the selected *A. lineatus* genes, motif enrichment analysis was conducted using the Analysis of Motif Enrichment (AME) tool implemented in MEME Suite v5.5.9 (https://meme-suite.org/meme/) and the JASPAR CORE 2024 vertebrate motif database. Enrichment was evaluated using Fisher’s exact test and a maximum odds score sequence-scoring approach. The promoter regions of the remaining species-specific genes identified through the orthology analysis of *A. lineatus* were used as the background set.

### Code Availability

No custom code was used in this study. All data processing was carried out using standardized pipelines of each free access software. The software package and versions for all the analyses are shown in Supplementary Tables 1 and detailed in the Methods section. For software tools where specific parameters are not explicitly mentioned, default settings provided by the developers were used.

## Results

### Genome Sequencing and *De novo* Assembly of Genome and Mitogenome

**Fig. 2:**
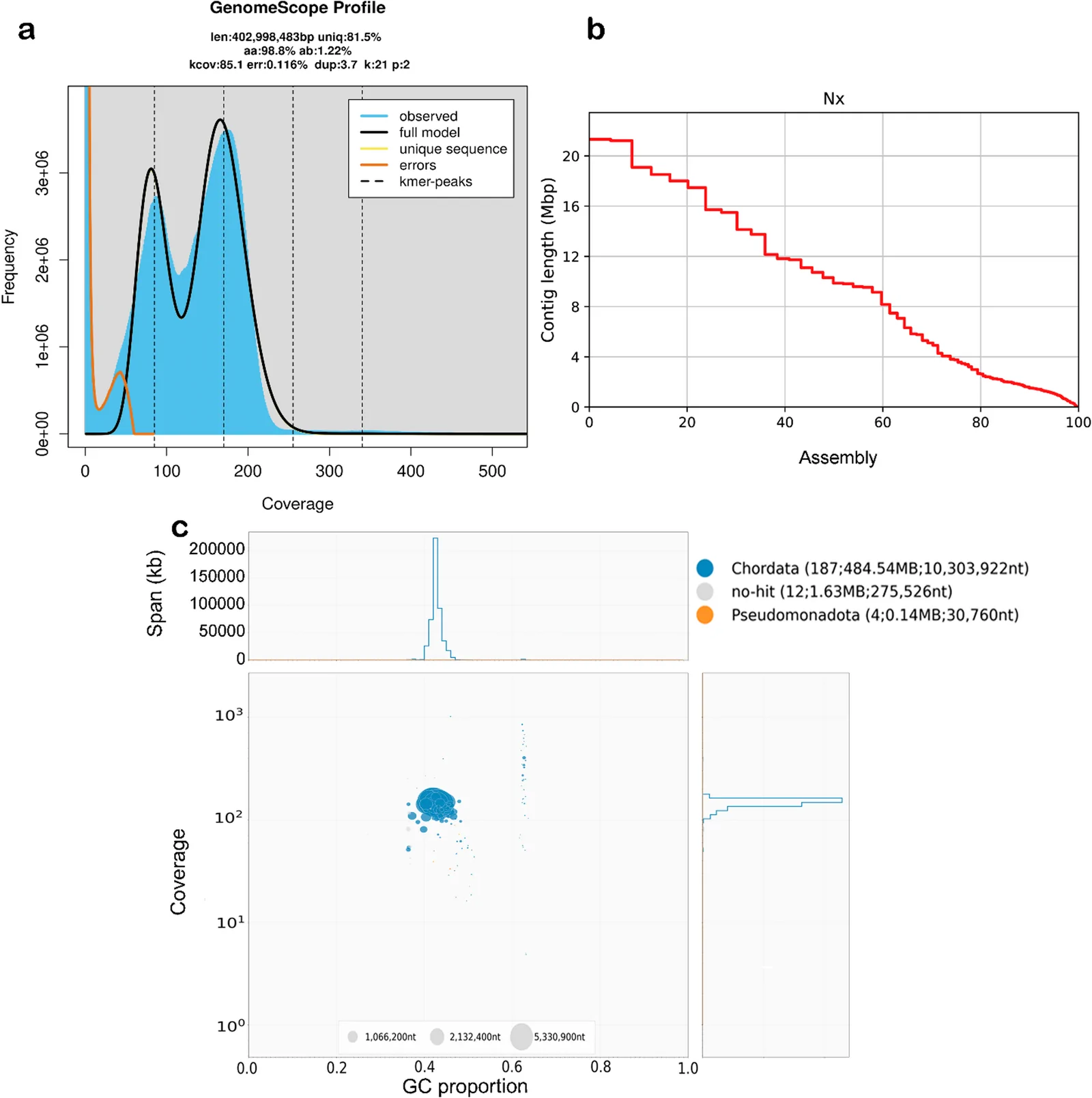
Genome assembly overview of *Achirus lineatus*. (**a**) K-mer frequency distribution plot of *A. lineatus* genome; len (length), uniq (unique k-mers), het (heterozygosity), kcov (k-mer coverage), err (error), dup (duplication), k (k-mer length). (**b**) Cumulative contig length Nx curve. (**c**) Taxon-annotated GC-coverage plot (BlobPlot)

PacBio long-read Whole‑Genome sequencing (WGS) generated 132 Gb of HiFi raw reads (~ 175× coverage), with a quality value (QV) greater than 20. After quality filtering, 71 Gb of ultra–high-quality HiFi reads (QV > 30 and read length > 10,000 bp) were retained, corresponding to approximately 93× coverage of the estimated *A. lineatus* genome size (Table 1). The k-mer analysis estimated a genome size of approximately 400 Mb, comprising 81.5% unique sequences and 18.5% repetitive content. The inferred heterozygosity rate was 1.22%, and the estimated sequencing error rate was low (0.116%). The presence of a dominant k-mer peak at approximately 85× coverage indicates sufficient sequencing depth and supports the generation of a high-quality *de novo* genome assembly (Fig. 2a).

**Table 1:** Statistics of sequencing data for *Achirus lineatus* genome assembly

PacBio HiFi library	Total Gb	HiFi reads	HiFi read bases	Average length (bp)	Largest read (bp)	Shortest read (bp)	Average read Quality	Coverage
Raw data	132	5,356,766	70,360,384,481	13,134	42,235	66	Q30	~ 175 ×
Clean data	71	2,796,581	37,637,981,512	13,458	30,841	10,000	Q34	~ 93 ×

A total of 203 non-redundant contigs were initially assembled. Cross-contamination assessment indicated that 93.46% of the sequences were assigned to Chordata, 6.54% had no taxonomic assignment, and only 0.01% were classified as Pseudomonadota (Fig. 2c). The latter sequences were eliminated before the subsequent analyses. As a result, the final genome assembly comprised of 199 contigs, with a total length of 486.17 Mb (Fig. 2b), a contig N50 of 9,865,541 bp, and a maximum contig length of 21,323,396 bp (Table 2). Assembly statistics also indicate high contiguity, with half of the genome contained within only 17 contigs (L50 = 17), and 90% of the genome represented by 67 contigs larger than 1,500,059 bp.

Quality assessment of the final assembly revealed high completeness, accuracy and consistency, with a BUSCO score of 99.2% (Table 2), a Merqury consensus QV of 59.9 and a base error rate of 1.00878 × 10^− 6^. The accuracy of the assembly was also confirmed by a high read mapping rate of 99.94% of raw reads successfully mapped back to the assembly. All metrics satisfy the quality standards established by the Earth BioGenome Project (EBP Sequencing and Assembly Standards Committee, January 31, 2026) (Lewin et al. 2018).

**Table 2:** Assembly statistics of *Achirus lineatus*

Assembly	*Achirus lineatus*
Total length	486.17 Mb
Contig number	199
Largest contig	21,323,396
Contig N50	9,865,541
L50	17
L90	67
auN	10,294,398
# N’s per 100 kbp	0
total N’s	0
GC (%)	42.76
Complete BUSCOs *	99.20%
Fragmented	0.50%
Missing	0.30%
Duplicated	0.70%

*BUSCO actinopterygii_odb10 lineage dataset

**Fig. 3:**
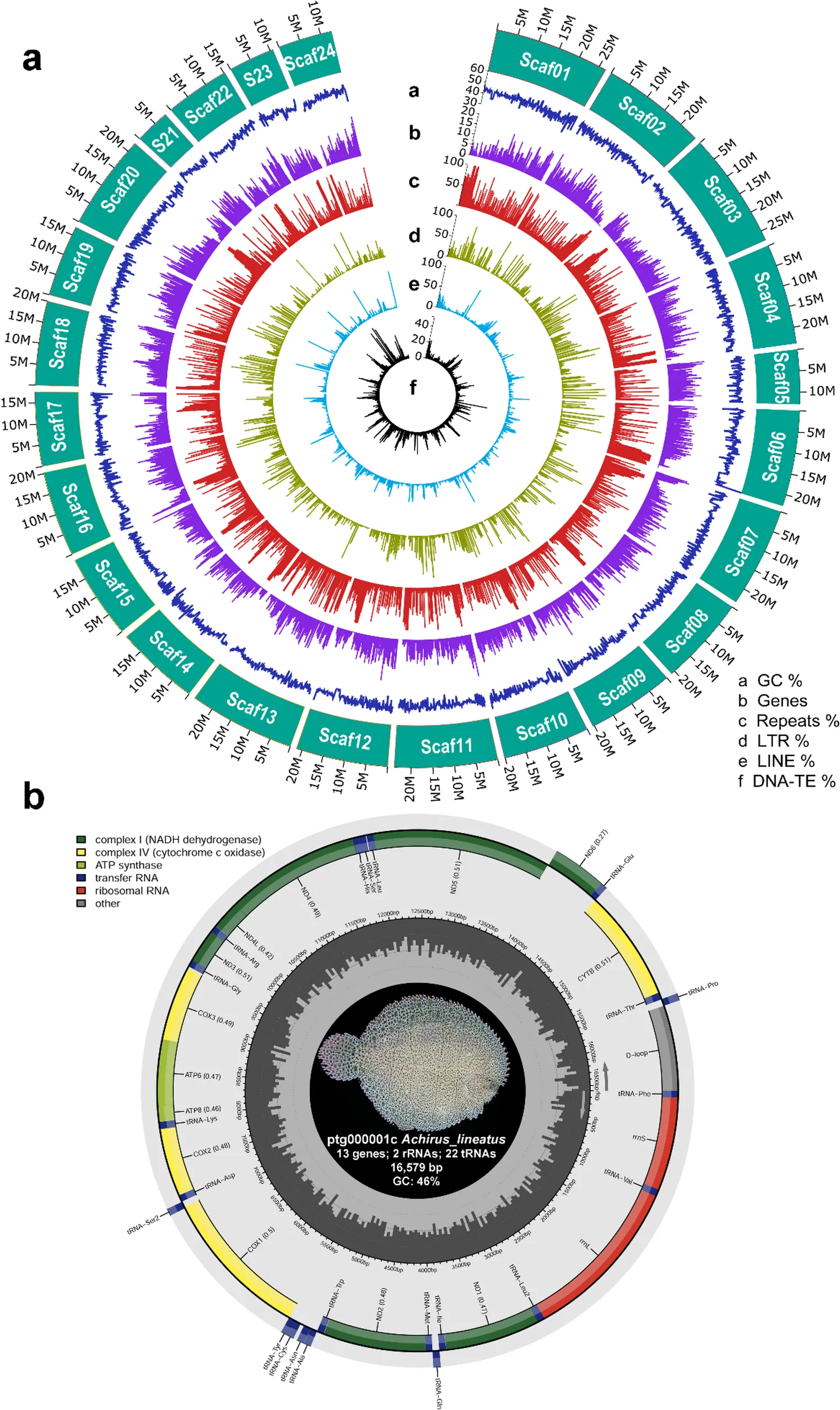
Genomic features visualization of *Achirus lineatus*. (**a**) Circos plot of reference-guided scaffold assembly. Tracks from outer to inner show GC content, distribution of gene counts, total repeat density, LTR density, LINE density, and DNA transposon density in 100 kb windows across the 24 largest scaffolds. For each track, the y-axis indicates respective observed ranges. (**b**) Circular representation of the mitochondrial genome. The mitogenome is 16,579 bp in length and contains 13 protein-coding genes (PCGs), 22 transfer RNA (tRNA) genes, 2 ribosomal RNA genes (rrnS [12 S ribosomal RNA] and rrnL [16 S ribosomal RNA]), and a single putative control region. Genes and RNAs are shown on the outer ring, with those transcribed clockwise located on the forward strand (inner features) and those transcribed counterclockwise on the reverse strand (outer features) and are color-coded by functional category. The inner histogram represents GC content of a given 100 bp interval across the mitogenome, with concentric rings indicating 25% increments

For the mitogenome assembly, a circular mitochondrial genome of 16,579 bp was obtained, with an average read depth of 854× based on mapped reads, supporting the robustness of the assembly. The final annotation identified 13 protein-coding genes, 2 rRNA genes, and 22 tRNA genes, with an overall GC content of 46% (Fig. 3b), consistent with the typical mitochondrial genome organization observed in other teleost fishes (Valencia-Pesqueira et al. 2025). A nucleotide BLAST search of the complete mitochondrial genome against the NCBI database (core-nt) revealed 99.28% sequence identity with previously deposited mitogenome of *A. lineatus* (accession NC_023768.1) (Shi et al. 2018). In addition, the Cytochrome c oxidase subunit I (COI) region showed a 100% match against the Barcode of Life Data System (BOLD) database, providing strong support for species-level identification.

### Genome Annotation and Scaffolding

Approximately 27.1% of the *A. lineatus* genome corresponds to repetitive elements, with LTR retrotransposons representing the most abundant annotated class (5.82%), followed by LINEs (2.95%) and DNA transposons (2.66%). A substantial fraction (10.76%) of repeats remained unclassified (Fig. 3a; Supplementary Table 2).

A total of 22,412 protein-coding genes were predicted. These genes exhibited an average length of 9,286 bp, with a mean coding sequence (CDS) length of 1,775 bp. On average, each mRNA contained 11 exons and 10 introns (Supplementary Table 3). The refinement with PASA (MAKER+PASA) resulted in modest improvements and provided additional support and validation for the final annotation. This refinement improved exon–intron boundary definition, enhanced untranslated region (UTR) annotation, and identified more complete coding sequences. The final annotation consisted of 22,100 gene models with an average gene length of 10,539 bp and a mean CDS length of 1,780 bp (Supplementary Table 4).

As an additional independent validation of the structural annotation, gene models predicted by the evidence-integrated MAKER pipeline and the refined MAKER+PASA annotation were compared with predictions generated by Helixer, a deep learning-based ab initio gene prediction tool. Comparable gene structure statistics were observed among these approaches (Supplementary Table 4). A total of 21,273 MAKER gene models (~ 95% of the predicted gene set) were shared with Helixer predictions, while 21,808 gene models (97%) were shared between MAKER and MAKER+PASA annotations, further supporting the robustness and consistency of the structural annotation. This gene structural organization is consistent with patterns commonly reported for other teleost fish genomes (Wang et al. 2025).

The predicted proteome revealed that 89.4% of BUSCO genes were complete, with comparable completeness values observed for OMArk Teleostei hierarchical orthologous groups (HOGs; 88.79%). In addition, functional consistency assessment of the whole-proteome using OMArk indicated that 91.9% of the predicted proteins corresponded to the expected Teleostei protein repertoire (Table 3) corresponding to a 6.7.Q40 quality category for the predicted gene set under the standards established by the Earth BioGenome Project (EBP Sequencing and Assembly Standards Committee, January 31, 2026) (Lewin et al. 2018). Also, no evidence of cross-lineage contamination was detected in the proteome (Table 3).

**Table 3:** BUSCO and OMArk assessment results for the predicted proteome of *Achirus lineatus*

Type	Number	Percentage (%)
BUSCO completeness assessment (actinopterygii_odb10)		
Total actinopterygii BUSCO	3,640	100
Complete BUSCOs	3,253	89.4
Single-copy	3,187	87.6
Duplicate	66	1.8
Fragmented	86	2.4
Missing	301	8.2
OMArk completeness assessment		
Total teleostei HOGs	14,195	100
Conserved HOGs	12,604	88.79
Single conserved	11,809	83.19
Duplicated conserved	795	5.60
OMArk whole proteome consistency assessment		
Number of proteins in the whole proteome	22,100	100
Associated query proteins in Teleostei	20,297	91.84
Consistent lineage placement (Teleostei)	20,066	90.80
Inconsistent lineage placement	231	1.05
Contamination	0	0
Total Unknown	1,803	8.16

To further evaluate gene annotation quality, the length distributions of gene features (CDS, exons, and introns) were compared with those of closely related species (*P. olivaceus*, *C. semilaevis*, *H. hippoglossus*, *H. stenolepis* and *S. solea*), revealing highly similar patterns across species and providing additional support for the accuracy of the gene prediction pipeline (Supplementary Fig. 1). In addition, the number of predicted mRNAs (longest isoform per gene) was compared among closely related species, and functional annotation was performed for all datasets under the same methodological framework. Comparable ranges were observed for both the number of predicted genes and the proportion of functionally annotated genes across species (Supplementary Table 7).

**Fig. 4:**
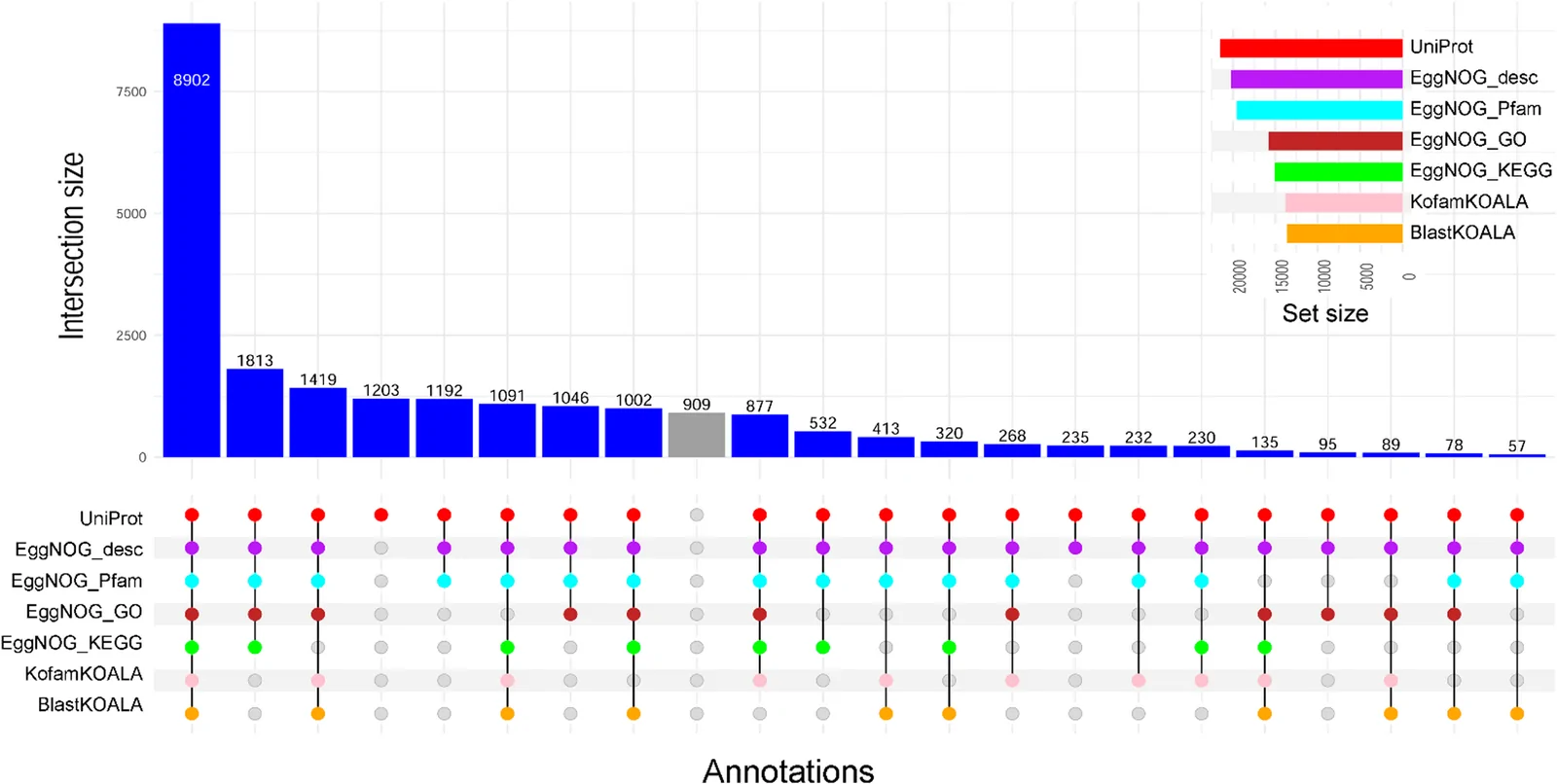
Functional annotation and collinearity analysis. UpSet plot of the functional annotation of the *A. lineatus* genome. The plot integrates seven functional annotation sources using five public databases: UniProt–UniRef90, Pfam (via EggNOG), GO (via EggNOG), KEGG (via EggNOG and BlastKOALA), and KOfam. EggNOG_desc stands for the general functional annotation obtained from EggNOG-mapper. The genes observed in the grey bar (909 genes) did not have functional annotations across the databases used

For functional annotation, 21,503 genes (95.94% of the predicted gene set) were successfully annotated through the integration of multiple methods and databases. BLASTp searches against UniProt–UniRef90 provided the highest annotation coverage (95.79%), followed by eggNOG-mapper (90.23%), whereas BlastKOALA (KEGG-based annotation) yielded the lowest number (60.72%) of annotated genes (Supplementary Table 5). Figure 4 shows an UpSet plot summarizing the overlap of functional annotations among UniProt–UniRef90, Pfam, Gene Ontology (GO), and KEGG databases. Only 909 genes (4.06%) lacked functional annotation across all databases used in this study.

Twenty-four reference-guided scaffolds were generated using the chromosome-level genome of *P. olivaceus* as a reference. These scaffolds do not necessarily represent the native chromosome number of *A. lineatus* (2n = 40; *n* = 20) but provide a useful framework for large-scale genomic visualization and feature distribution analyses (Fig. 3a). A total of 476,743,491 bp of the *A. lineatus* genome were distributed in the resulting scaffolds, with a mean length of 19,864,312 bp per scaffold (Fig. 3a). These represent approximately 98% of the total *A. lineatus* genome assembly, whereas 67 contigs totaling 9,407,029 bp (1.93%) remained unplaced.

The reference-guided scaffold assembly introduced only 10,700 ambiguous bases (2.2 Ns per 100 kb), distributed across 287 gaps with an average gap size of 37 bp and a maximum gap size of 60 bp, indicating minimal unresolved regions after scaffolding.

### Comparative Genome Analysis

**Fig. 5:**
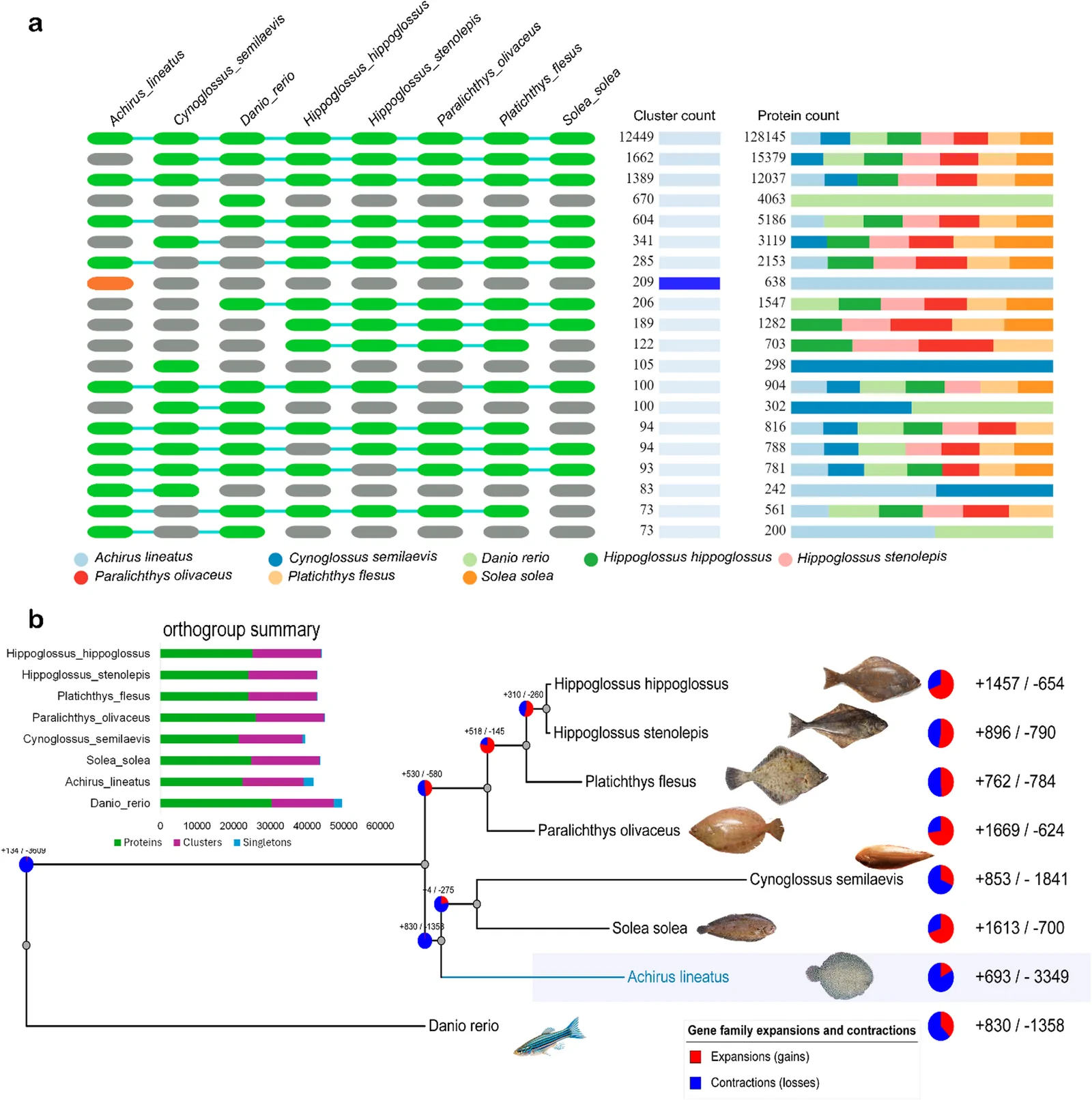
Orthology analysis and phylogenetic relationships among *Achirus lineatus*, *Danio rerio*, and six other flatfish species (*C. semilaevis*, *S. solea*, *P. olivaceus*, *P. flesus*, *H. hippoglossus*, *H. stenolepis*). (**a**) Orthology clusters and total proteins shared among *A. lineatus* and the other seven fish species. (**b**) Orthogroup summary and gene family expansions and contractions constructed from the orthology analysis

A total of 20,760 gene families (orthogroups) were identified across the 8 species analyzed, encompassing approximately 96.6% of all genes across the species (Supplementary Table 6), while the remaining 3.4% were not assigned to any orthogroup. Figure 5a shows the distribution and overlap of orthologous gene clusters across all analyzed species, highlighting that 12,449 orthogroups were shared across all the species while *A. lineatus* possesses 209 species-specific gene families.

Using 4,202 single-copy orthologous genes from the concatenated alignment generated by OrthoFinder (Supplementary Table 6). A maximum likelihood (ML) phylogenetic tree with the JTT + F+R4 substitution model was constructed to infer evolutionary relationships among the analyzed species. *A. lineatus* clustered within the pleuronectiform lineage and was phylogenetically closest to *S. solea* and *C. semilaevis*, forming a distinct clade separated from the Paralichthyidae/Pleuronectidae group represented by Paralichthys, Platichthys, and Hippoglossus species. The two Hippoglossus species formed a well-supported monophyletic clade, and all internal nodes showed maximal branch support (SH-aLRT/UFBoot = 100/100) (Fig. 5b).

For the expansion–contraction analysis, model comparison supported a uniform birth–death model across branches, with a global gene gain/loss rate of λ = 8.96 × 10^− 4^. *A. lineatus* exhibited 693 expanded and 3,349 contracted gene families (Fig. 5b), indicating a predominance of gene family contraction in *A. lineatus*. Expanded gene families showed significant GO enrichment in biological processes related to synapse organization, neuron differentiation, generation of neurons, cell junction organization, neurogenesis, neuron development, neuron projection development, nervous system development, regulation of neuron differentiation, and cell junction assembly, among others (Supplementary Fig. 2; Online Resource 1). Consistent enrichment patterns were also observed for molecular function and cellular component categories, indicating that expanded gene families in *A. lineatus* are primarily associated with neuronal development and connectivity, synaptic plasticity, and neuronal signaling (Supplementary Fig. 2).

In contrast, contracted gene families were significantly enriched in biological processes associated with myeloid leukocyte mediated immunity, leukocyte degranulation, myeloid cell activation involved in immune response, neutrophil mediated immunity, neutrophil degranulation, granulocyte activation, neutrophil activation involved in immune response, myeloid leukocyte activation, leukocyte mediated immunity, among others (Supplementary Fig. 3; Online Resource 2).

### Identification of Xenobiotic Metabolism Pathways

The genome can respond to chemical exposure through several mechanisms that protect the organism against chemical pollution, helping to maintain fitness and adapt to overcome the exposure effect. One of the main molecular pathways involved in the biotransformation of pollutants and the reduction of cellular toxicity is the xenobiotic metabolism pathway.

To further investigate this pathway, the genomes of *A. lineatus*, *C. semilaevis*, *D. rerio*, *H. hippoglossus*, *H. stenolepis*, *P. olivaceus*, *P. flesus*, and *S. solea* were annotated following the same methodological framework to obtain KEGG annotations and enable a comparative analysis of the xenobiotic biodegradation and metabolism gene repertoire among species.

The KEGG-based annotation revealed a total of 123 genes assigned to xenobiotic biodegradation and metabolism pathways in *A. lineatus* (Online Resource 3 and 4). Comparable numbers of genes associated with these pathways were observed across the other analyzed species (Supplementary Table 8), with *D. rerio* showing the highest number of xenobiotic metabolism-related genes (235 genes). Across all species, gene families involved in Phase I xenobiotic biotransformation (e.g., cytochrome P450 monooxygenases, dehydrogenases, and hydrolases) were more abundant than those associated with Phase II biotransformation processes (e.g., glutathione S-transferases and UDP-glucuronosyltransferases).

**Fig. 6:**
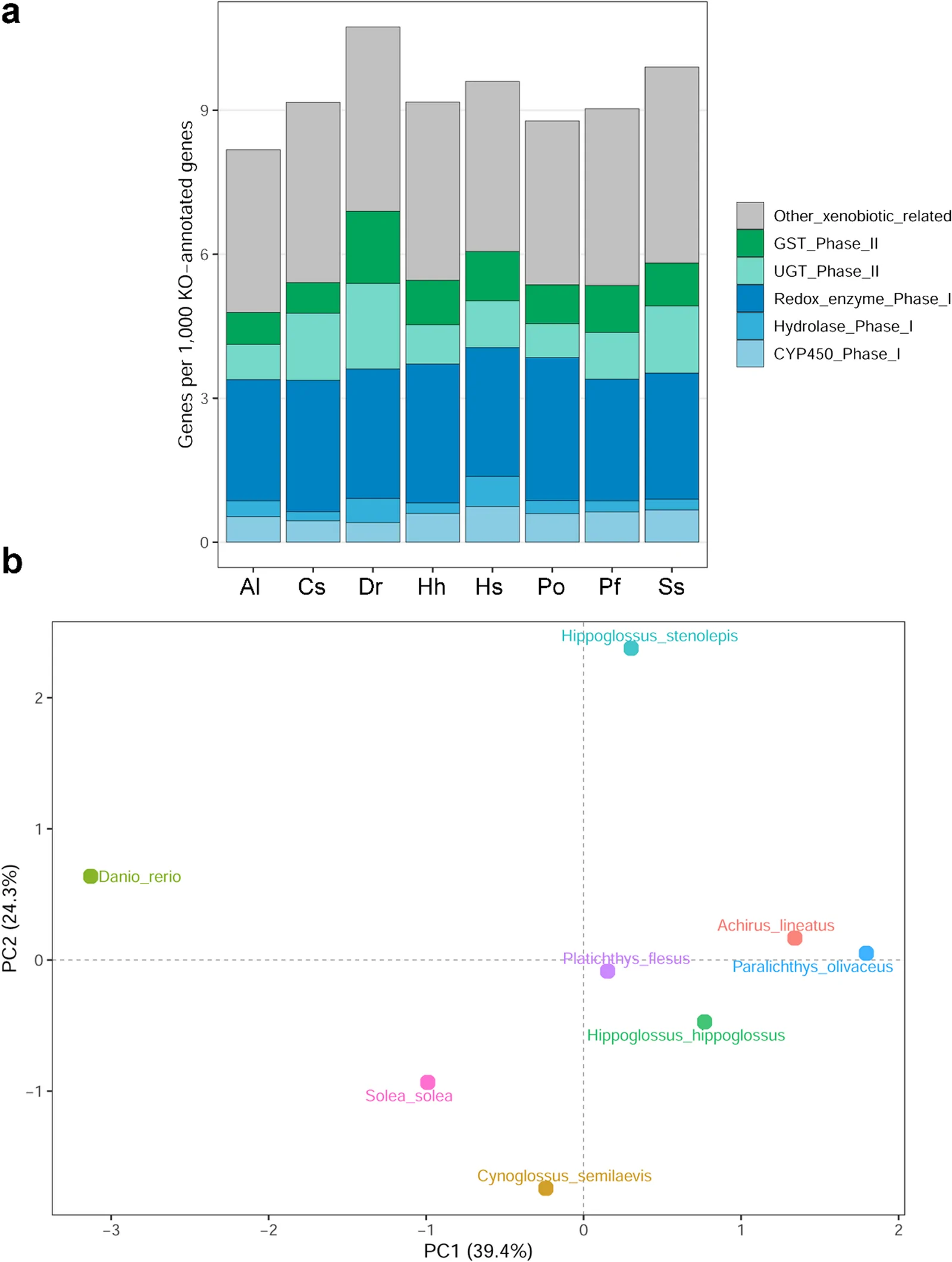
Comparative biotransformation gene families involved in xenobiotic biodegradation and metabolism in *Achirus lineatus* and seven other species. (**a**) Stacked barplot showing normalized counts of gene families annotated in xenobiotic biodegradation and metabolism pathways. Values are expressed as genes per 1,000 KO-annotated genes. CYP450 (Phase I) cytochrome P450; Hydrolase (Phase I): esterases and hydrolases; Redox enzyme (Phase I): dehydrogenases, reductases, oxidases and dioxygenases; UGT (Phase II): UDP-glucuronosyltransferase; GST (Phase II): glutathione S-transferase. AL: *Achirus lineatus*, Cs: *Cynoglossus semilaevis*, Dr: *Danio rerio*, Hh: *Hippoglossus hippoglossus*, Hs: *Hippoglossus stenolepis*, Po: *Paralichthys olivaceus*, Pf: *Platichthys flesus*, Ss: *Solea solea*. (**b**) Principal component analysis (PCA) based on normalized gene families across species

Comparative analyses further revealed conserved distributions of both Phase I and Phase II detoxification-related gene families among species (Fig. 6a). Consistent with this pattern, principal component analysis clustered *A. lineatus* within the variation observed among flatfish species, whereas *D. rerio* displayed a more distinct profile (Fig. 6b). Fisher’s exact tests detected no significant enrichment of xenobiotic metabolism categories in *A. lineatus* after false discovery rate (FDR) correction.

**Fig. 7:**
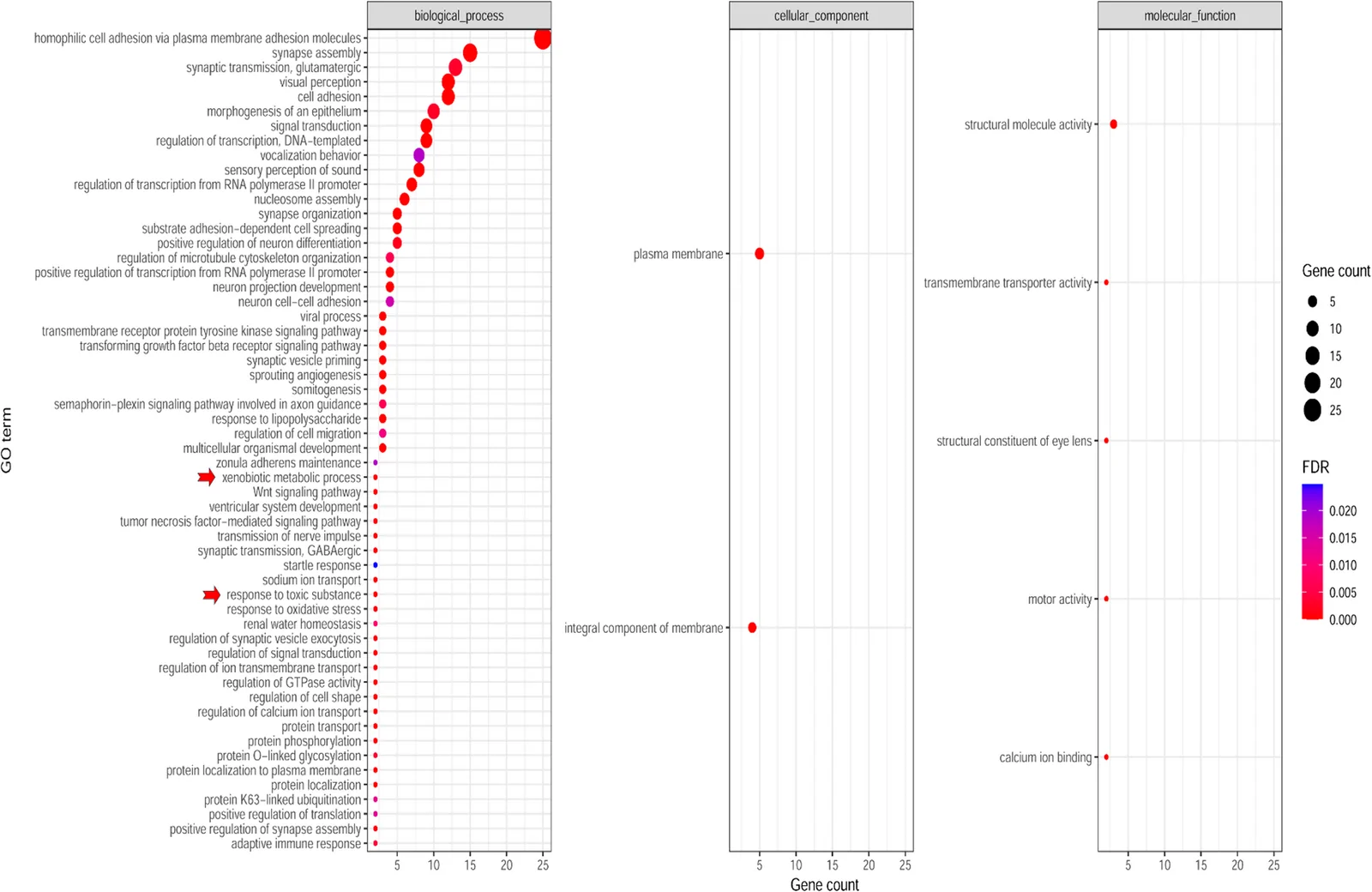
Enriched GO terms in orthologous gene clusters specific to *A. lineatus* (*p*_adj_ < 0.05). The red arrows indicate biological processes associated with environmental toxicology

**Fig. 8:**
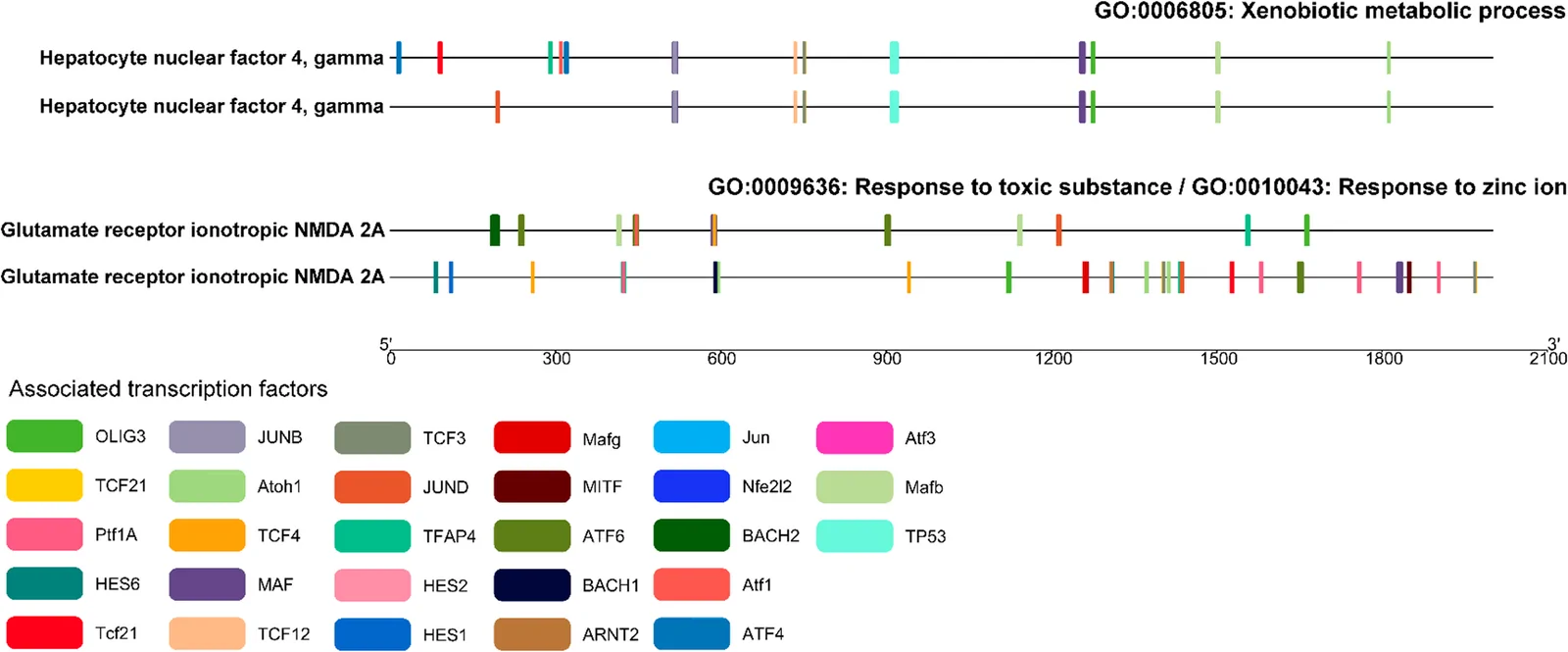
*Cis*-regulatory elements in promoter region of orthologous gene clusters specific to *Achirus lineatus* related to xenobiotic metabolism and response to toxic substances. The transcription factors associated with each motif were annotated in the *A. lineatus* genome

Moreover, from the species-specific genes identified in *A. lineatus*, a total of 209 gene clusters, representing 638 proteins, were subjected to GO enrichment analysis to characterize species-specific functional signatures (Fig. 7; Online Resource 5). This analysis revealed a significant enrichment of biological processes related to environmental toxicology, including xenobiotic metabolic processes and responses to toxic substances (Fig. 7). Their potential implication for chemical stress response and detoxification is a topic that deserves exhaustive investigation. Thus, to explore the regulatory basis of these patterns, we analyzed the promoter regions of these genes to identify *cis*-regulatory elements (CREs) potentially involved in their transcriptional control. Additionally, the presence of the corresponding transcription factors was examined within the genome to evaluate their potential regulatory relevance (Fig. 8).

The promoter regions of selected *A. lineatus*–specific genes involved in xenobiotic metabolism and responses to toxic substances exhibited a high density and diversity of *cis*-regulatory elements (CREs), indicating a complex transcriptional regulatory landscape. In the case of hepatocyte nuclear factor 4 gamma, both gene copies displayed highly similar promoter architectures, with only minor divergence at the 5′ end of the promoter region (Fig. 8). In contrast, the two copies of the glutamate receptor ionotropic NMDA 2 A gene showed markedly divergent promoter regions, characterized by a higher number and greater diversity of CREs, suggesting a more complex and potentially multilayered regulatory control (Fig. 8).

Several of the identified CREs corresponded to transcription factors implicated in oxidative stress, detoxification, chemical stress, and general environmental stress–response pathways, including NFE2L2, ATF3, ATF4, ATF6, ARNT2, and TP53. The presence of these regulatory elements in the *A. lineatus* genome supports the existence of a dynamic and potentially inducible transcriptional regulatory framework, consistent with the involvement of these genes in cellular responses to environmental and chemical challenges.

The promoter regions of the four xenobiotic-associated species-specific genes were evaluated for transcription factor binding motif enrichment relative to the remaining species-specific genes. Although several putative transcription-factor-binding motifs were previously identified through FIMO analysis and supported by proteome annotation, AME did not detect statistically significant motif enrichment relative to the background set after correction for multiple testing.

## Discussion

Flatfish have been reported as sensitive sentinels for environmental disturbances associated to pollutants (Dierking et al. 2009; Mirbahai et al. 2013; Rocha et al. 2014; Brown-Peterson et al. 2015, 2017; Jung et al. 2018; Quintanilla-Mena et al. 2020; Partheeban et al. 2021; Roubeix et al. 2023; Sepp et al. 2024; Cohen-Sánchez et al. 2025), and are widely used in biomonitoring programs worldwide, including those under the frameworks of the OSPAR Commission and the International Council for the Exploration of the Sea (Mirbahai et al. 2013; Roubeix et al. 2023).

In the western Atlantic, the lined sole *A. lineatus* is a suitable sentinel species for biomonitoring and ecological studies due to its wide distribution, ease of sampling, and biological characteristics (de Oliveira and Fávaro 2010; Améndola-Pimenta et al. 2020; Roubeix et al. 2023). Additionally, this species can be maintained successfully in captivity under controlled laboratory conditions, and its sensitivity to petroleum-derived contaminants has been demonstrated following exposure to oil Water Accommodated Fraction (WAF) (Améndola-Pimenta et al. 2020; Cerqueda-García et al. 2020; Zamora-Briseño et al. 2021; Cañizares-Martínez et al. 2024a). Nevertheless, the limited genomic data available for *A. lineatus* has been a weakness to consider it as a model species. The integration of omics approaches, including whole-genome sequencing and annotation, is essential to continue investigating pollutant-associated pathways.

In this study, we present a high-quality draft genome assembly and annotation of *A. lineatus*, generated using PacBio HiFi long-read sequencing and supported by previously published transcriptomic data (Zamora-Briseño et al. 2021). The high coverage of clean sequencing data (93×) allowed the generation of a high-quality, well-annotated genome (Tables 2 and 3; Fig. 4), comparable to those reported for chromosome-level fish genomes generated through integrative approaches combining long-read sequencing platforms (PacBio or Oxford Nanopore) with Hi-C scaffolding techniques (Gao et al. 2024; Sun et al. 2024; Zhou et al. 2024; Wang et al. 2025, 2026a, b; Valencia-Pesqueira et al. 2025; Liu et al. 2026; Marcuzzo et al. 2026; Zhang et al. 2026).

To further validate assembly quality, the raw RNA-seq reads used as evidence for genome annotation were mapped against the assembly, resulting in more than 90% uniquely mapped reads. The genomic profile and predicted gene repertoire obtained in this study also agree with genomic features typically observed in flatfish species (Lü et al. 2021).

*A. lineatus* genomic profile showed a high heterozygosity rate (1.22%) (Fig. 2a), inferred from the k-mer frequency distribution, suggesting substantial genetic diversity that could be related to adaptation to heterogeneous environments (Mwamburi et al. 2024). The coastal lagoons where this species is commonly distributed present temporal and spatial fluctuations in environmental parameters such as salinity and temperature (Kuk-Dzul et al. 2012; Chuang et al. 2017). This genetic diversity could be associated with the organisms’ capacity for environmental adaptation that may facilitate responses to heterogeneous and dynamic ecological conditions (Yan et al. 2025). Further population-level genetic analyses, such as Simple Sequence Repeats (SSRs) or Single Nucleotide Polymorphisms (SNPs) with a larger number of samples, will be necessary to test this hypothesis in the lined sole.

The DNA extracted from liver and gill tissues resulted in high-quality sequencing data. Although gills are in more direct contact with the environment and could potentially introduce microbial contamination, the quality-control and cross-contamination analyses conducted in this study revealed minimal evidence of microbial contamination (Fig. 2c). The taxon-annotated GC-coverage plot also showed a small number of contigs with higher GC proportions (Fig. 2c), which likely reflect natural intragenomic compositional heterogeneity rather than contamination. In vertebrates, GC-rich genomic regions are often associated with coding-rich segments or compositionally distinct genomic domains. Similar patterns have been reported in the sea lamprey genome, where protein-coding regions reached GC values above 60% (Smith et al. 2013).

Regarding to the repetitive landscape, the annotation of *A. lineatus* is comparable to that reported for other fish genomes (Lü et al. 2021; Sun et al. 2024; Zhou et al. 2024). Notably, the fraction of unclassified repeats detected in this study (10.76%), is consistent with observations in teleost genomes, where a substantial proportion of repetitive elements often remains uncharacterized due to high lineage-specific divergence and inherent limitations of homology-based repeat annotation approaches (Lü et al. 2021; Reinar et al. 2023).

For structural gene prediction, we identified 22,412 protein-coding genes, a number that is consistent with those reported in other flatfish genomes (Lü et al. 2021). But the BUSCO completeness score for the predicted proteome was lower than that obtained for the genome assembly (Tables 2 and 3). Such discrepancies between genome- and proteome-based BUSCO assessments have been reported in other genomic studies and are commonly attributed to limitations of *de novo* gene prediction algorithms (Saary et al. 2020; Mwamburi et al. 2024). Nevertheless, the evaluation of the complete predicted proteome using OMArk, in relation to the expected protein repertoire in teleost revealed a high proportion of expected proteins (91.9%), supporting the overall quality of the predicted protein set. Furthermore, 95.9% of this gene set was functionally annotated, providing additional evidence of the robustness and completeness of the proteome annotation (Fig. 4).

To obtain a biologically relevant comparison and improve chromosome-scale inference in *A. lineatus*, we used the chromosome-level genome of the Japanese flounder (*P. olivaceus*) as a reference for pseudochromosomes construction. *P. olivaceus* is an economically important species in China, Korea, and Japan, where intensive aquaculture has supported the development of a high-quality chromosome-level reference genome (Xu et al. 2022; Li et al. 2025). Also, this species has been widely used as a model in toxicological and stress-response studies (Song et al. 2012; Zhu et al. 2016; Jung et al. 2018; Xu et al. 2022; Li et al. 2025), making its genome a robust and biologically meaningful reference for collinearity analyses.

Though the haploid chromosome number of *P. olivaceus* is 24, the haploid chromosome number reported for *A. lineatus* is 20 (Carvalho De Azevedo et al. 2005), but the proportion of unplaced contigs remained low (1.93%). And most of the *A. lineatus* genome assembly were successfully aligned to *P. olivaceus* chromosomes (98.6%), further supporting the suitability of this reference genome for scaffolding and genome completeness comparisons.

Another aspect contributing to adaptation, evolution, and functional diversification in species is the expansion and contraction of gene families (Wang et al. 2025). The comparative genomic analysis conducted here revealed that a high proportion of *A. lineatus* genes (87%) were assigned o orthogroups shared among all fish species included in the analysis, indicating a robust comparative framework (Fig. 5a; Supplementary Table 6) (Mwamburi et al. 2024). Nevertheless, *A. lineatus* exhibited the lowest number of gene family expansions (+ 693) and the highest number of gene family contractions (− 3,349) among the analyzed species. Gene family reduction is a common evolutionary process in response to environmental changes (Olson 1999); therefore, the high number of contracted gene families observed in *A. lineatus* may be associated with environmental adaptation to highly dynamic habitats such as coastal lagoons. This is a topic that deserves further investigations.

However, these results should be interpreted cautiously, as gene family contraction estimates may be influenced not only by true gene loss events but also by phylogenetic sampling and lineage representation in comparative analyses. In particular, the limited availability of closely related species may affect orthogroup reconstruction and ancestral gene family size estimation (Eirín-López et al. 2012). Furthermore, annotation heterogeneity across diverse genomic pipelines can exacerbate these inaccuracies, frequently leading to the misidentification of lineage-specific gene losses (Domazet-Lošo et al. 2024).

On the other hand, gene family expansion is generally associated with functional innovation (Olson 1999; Wang et al. 2025). In *A. lineatus*, we observed a consistent and significant expansion of gene families related to the nervous system and synaptic function, including synapse organization, neuron differentiation, generation of neurons, nervous system development, and regulation of neuron differentiation. This pattern suggests an expansion of neuronal plasticity and indicates an important adaptation of the nervous system. These findings align with comparative genomic surveys identifying dynamic gene family expansions in neural-associated pathways as a primary driver of lineage-specific physiological innovation (Albertin et al. 2015; Cicconardi et al. 2023).

The term “chemical defensome” is widely used to refer to molecular defenses against contaminants and involves genes and proteins, that help reduce chemical toxicity and eliminate harmful substances, grouped into four main functional categories: transcription factors (e.g., nuclear receptors), biotransformation pathways (e.g., phase I and II enzymes), transporters (ATP-dependent proteins), and stress responses (e.g., antioxidant proteins) (Eide et al. 2021; Franco et al. 2025). The composition of genes involved in these mechanisms in each species affects their sensitivity to contaminated environments (Franco et al. 2025).

To get a better understanding of the chemical defensome composition in *A. lineatus*, a comparative functional annotation analysis was conducted across several flatfish species, using *D. rerio* as an outgroup. Emphasis was placed on genes involved in biotransformation pathways, given the long-term historical exposure to petroleum hydrocarbons in the GoM (Pulster et al. 2020). A total of 123 genes in *A. lineatus* were assigned to KEGG Xenobiotics biodegradation and metabolism pathway (Supplementary Table 8). This number was comparable to those observed in the other flatfish species, whereas the outgroup exhibited a higher number of genes associated with this pathway (Supplementary Table 8). Comparative analyses based on stacked barplots and principal component analysis indicated that the overall repertoire of xenobiotic biodegradation genes is highly conserved among the analyzed species. This observation was further supported by the absence of significant differences in gene-category frequencies among species according to Fisher’s exact tests. Moreover, the PCA revealed an even higher degree of conservation among flatfish species, which clustered more closely to each other than to the outgroup (Fig. 6a, b).

These numbers are also consistent with reports for other teleost genomes, where detoxification-related gene families such as cytochrome P450s, glutathione S-transferases and UDP-glucuronosyltransferases are often expanded. Such expansions have been associated with adaptation to chemically variable or contaminated environments, particularly in coastal and estuarine fishes (Lee et al. 2018; Eide et al. 2021). However, the overall conservation observed in the xenobiotic biotransformation gene repertoire among the analyzed species may suggest that *A. lineatus* does not exhibit a uniquely specialized genomic composition regarding these pathways. Instead, adaptive responses to contaminant exposure could involve more complex molecular and regulatory interactions, including species-specific differences in gene regulation, expression dynamics, and sensitivity to xenobiotics. Nevertheless, further functional, experimental and comparative studies will be necessary to evaluate this hypothesis.

In particular, biotransformation pathways in fishes widely used as model species in toxicological studies (*D. rerio*, *Oryzias latipes*, *Fundulus heteroclitus*, and *Gasterosteus aculeatus*) or as bioindicator species in environmental monitoring programs (*Gadus morhua*) show a similar gene family composition for genes encoding *GST*s (9 to 19 genes) and *UGT*s (11 to 31 genes) (Eide et al. 2021), compared with those found in this study (Supplementary Table 8). Although we found an overall concordant number of genes involved in xenobiotic metabolism, variation in the composition of other gene families among species may be related to species-specific sensitivity or tolerance to chemical contamination (Eide et al. 2021; Lawrence et al. 2022).

Furthermore, the evaluation of additional pathways potentially associated with species-specific sensitivity, including disease-related pathways, could provide a more comprehensive understanding of responses to contaminant exposure. A large proportion of the KEGG pathways annotated in this study for *A. lineatus* were associated with disease-related pathways (Online Resource 4). However, these pathways are commonly linked to highly conserved molecular and cellular processes, and their enrichment may reflect functional homology rather than the presence of pathological conditions. In particular, the enrichment of genes in KEGG disease-related pathways such as cancer and neurodegenerative diseases is driven by the high conservation and pleiotropic roles of genes involved in fundamental cellular processes, including signal transduction, stress response, apoptosis, and mitochondrial function. Many of these conserved pathways are also implicated in cellular responses to environmental stress and toxicant exposure, highlighting their relevance in toxicogenomic and environmental adaptation studies (Hotamisligil and Davis 2016).

The presence of gene families unique to *A. lineatus* (638 proteins distributed across 209 orthogroups) suggests lineage-specific genomic innovation (Fig. 5a). A subset of these unique gene families is enriched in biological processes related to xenobiotic metabolism and responses to toxic substances (Fig. 7; Online Resource 5), indicating that part of this genomic novelty may contribute to adaptation to chemically variable environments. Among the genes contributing to this enrichment are hepatocyte nuclear factor 4 gamma (*HNF4G*), a nuclear receptor transcription factor involved in the regulation of lipid metabolism, xenobiotic-responsive genes, and hepatic and intestinal functions (Sasaki et al. 2018), and glutamate receptor ionotropic NMDA 2 A (*GRIN2A*), a key component of excitatory synaptic transmission associated with synaptic plasticity and known to be sensitive to neurotoxic insults such as lead exposure (Wu et al. 2017; Yasmin et al. 2025).

The GO enrichment analysis observed in both; the expanded gene families and the species-specific families of *A. lineatus* is consistent (Supplementary Fig. 2; Fig. 7). Also, the involvement of metabolic detoxification regulators and neuroplasticity-related genes suggests an integrated adaptive strategy combining physiological detoxification and neural responsiveness, which may be particularly relevant for survival in dynamic and potentially contaminated habitats. For example, chemical-induced alterations in the olfactory sensory system have been associated with avoidance behavior in response to chemical contamination (Franco et al. 2025). Future analyses of gene expression may offer additional insights into the adaptive relevance (e.g. avoidance behavior) of these genes under different environmental conditions.

Furthermore, the detection of *cis*-regulatory elements (CREs) in the promoter regions of *HNF4G* and *GRIN2A* associated with transcription factors such as Nuclear Factor Erythroid 2–Like 2 (*NFE2L2)*, Aryl Hydrocarbon Receptor Nuclear Translocator 2 (*ARNT2)*, Activating Transcription Factors (*ATF3*, *ATF4*, ATF6), and Tumor Protein p53 (*TP53)* indicates that these genes are under tight transcriptional control and are responsive to oxidative, chemical, and environmental stress signals (Fig. 8).

*NFE2L2* is a well-established regulator of xenobiotic metabolism and oxidative stress responses, including the activation of detoxification genes under chemical exposure (He et al. 2020). ARNT2 is a transcriptional co-regulator that forms heterodimers with ligand-activated transcription factors, including the aryl hydrocarbon receptor (AhR). Although primarily described in environmental sensing and neurodevelopmental contexts, ARNT2 has also been associated with apoptosis and oxidative stress, suggesting that it could play a role in modulating transcriptional responses to environmental and chemical stimuli (Shahriar et al. 2025; Jin et al. 2025). Stress-responsive transcription factors such as *ATF3*, *ATF4*, and *ATF6* mediate transcriptional responses to extracellular stress signals, including oxidative and chemical stress (Hai et al. 1999; Kreß et al. 2023; Lee et al. 2023), while *TP53* integrates stress signaling with DNA damage responses (Zhang et al. 2024). This regulatory architecture supports the proposed involvement of these genes within the chemical defensome, contributing to detoxification and stress-response mechanisms (He et al. 2020; Eide et al. 2021; Franco et al. 2025).

Although not statistically significant CRE enrichment was detected, the presence of motifs associated with transcription factors annotated in the *A. lineatus* genome and known to be involved in chemical stress responses supports the hypothesis that these genes could be transcriptionally regulated as part of molecular response pathways activated upon contaminant exposure.

## Conclusions

The genomic resources generated for *A. lineatus* offer insights into molecular processes potentially involved in tolerance for chemical and physical stressors in dynamic habitats. Results from this study provide baseline genomic information that may support future evaluations of *A. lineatus* as a bioindicator species for assessing the ecological health of coastal lagoons in the GoM and other areas along its geographical distribution. Additionally, this study provides an important foundation for future ecotoxicogenomic, transcriptomic, epigenomic, and adaptive evolution studies in flatfishes, including the implementation of branch-site selection analyses and comparative evolutionary approaches under different environmental exposure scenarios and under controlled experimental designs.

## Supplementary Information

Below is the link to the electronic supplementary material.

Supplementary Material 1 (XLSX 29.7 KB)

Supplementary Material 2 (XLSX 27.3 KB)

Supplementary Material 3 (XLSX 967 KB)

Supplementary Material 4 (XLSX 25.1 KB)

Supplementary Material 5 (XLSX 13.2 KB)

Supplementary Material 6 (DOCX 1 MB)

## Data Availability

The sequencing data (filtered PacBio HiFi reads; Q > 30, read length > 10,000 bp), the genome assembly, and the annotation generated in this study are available in the NCBI database under BioProject accession number PRJNA1434538. The mitochondrial genome assembly and its annotation are available on GenBank accession number PZ155360. In addition, the genome assembly, masked genome assembly, and genome annotation files are available in the Figshare repository (DOI: 10.6084/m9.figshare.31796092).

## References

[CR1] Albertin CB, Simakov O, Mitros T, Wang ZY, Pungor JR, Edsinger-Gonzales E, Brenner S, Ragsdale CW, Rokhsar DS (2015) The octopus genome and the evolution of cephalopod neural and morphological novelties. Nature 524:220–224. 10.1038/nature14668 26268193 10.1038/nature14668 PMC4795812

[CR2] Aleksander SA, Balhoff JP, Carbon S, Cherry JM, Ebert D, Feuermann M, Gaudet P, Harris NL, Hill DP, Kalita P, Lee R, Mi H, Moxon S, Mungall CJ, Muruganujan A, Mushayahama T, Sternberg PW, Thomas PD, Van Auken K, Wong ED, Wood V, Ramsey J, Siegele DA, Chisholm RL, Dodson R, Fey P, Aspromonte MC, Nugnes MV, Naser XAC, Tosatto SCE, Giglio M, Nadendla S, Antonazzo G, Attrill H, Brown NH, dos Santos G, Marygold S, Röper K, Strelets V, Tabone CJ, Thurmond J, Zhou P, Zaru R, Lovering RC, Logie C, Chen D, Naba A, Christie K, Corbani L, Ni L, Sitnikov D, Smith C, Seager J, Cooper L, Elser J, Jaiswal P, Gupta P, Naithani S, Carme P, Rutherford K, De Pons JL, Dwinell MR, Hayman GT, Kaldunski ML, Kwitek AE, Laulederkind SJF, Tutaj MA, Vedi M, Wang S-J, D’Eustachio P, Aimo L, Axelsen K, Bridge A, Hyka-Nouspikel N, Morgat A, Goldbold G, Engel SR, Miyasato SR, Nash RS, Sherlock G, Weng S, Bakker E, Berardini TZ, Reiser L, Auchincloss A, Argoud-Puy G, Blatter M-C, Boutet E, Breuza L, Casals-Casas C, Coudert E, Estreicher A, Famiglietti ML, Gos A, Gruaz-Gumowski N, Hulo C, Jungo F, Mercier P, Le, Lieberherr D, Masson P, Pedruzzi I, Pourcel L, Poux S, Rivoire C, Sundaram S, Bateman A, Adesina A, Bowler-Barnett E, Carpentier D, Denny P, Ignatchenko A, Ishtiaq R, Lock A, Lussi Y, Magrane M, Martin MJ, Orchard S, Raposo P, Speretta E, Tyagi N, Urakova N, Warner K, Yu CW-H, Chan J, Diamantakis S, Quinton-Tulloch M, Raciti D, Fisher M, James-Zorn C, Ponferrada V, Zorn A, Howe D, Ramachandran S, Ruzicka L, Westerfield M (2026) The Gene Ontology knowledgebase in 2026. Nucleic Acids Res 54:D1779–D1792. 10.1093/nar/gkaf1292 41413728 10.1093/nar/gkaf1292 PMC12807639

[CR3] Alonge M, Lebeigle L, Kirsche M, Jenike K, Ou S, Aganezov S, Wang X, Lippman ZB, Schatz MC, Soyk S (2022) Automated assembly scaffolding using RagTag elevates a new tomato system for high-throughput genome editing. Genome Biol 23. 10.1186/s13059-022-02823-7 10.1186/s13059-022-02823-7 PMC9753292 36522651

[CR4] Amendola-Pimenta M, Aguirre-Macedo ML, Couoh-Puga ED, May-Tec AL, Quintanilla-Mena MA, Puch-Hau CA, Rodriguez-Gonzalez A, Vidal-Martinez VM, Rodriguez-Canul R, Pech D (2020) Vulnerabilidad de especies selectas de peces bentónicos y pelágicos expuestos a hidrocarburos de petróleo en condiciones experimentales. In: Aguirre-Macedo ML, Pérez-Brunius P, Saldaña-Ruiz LE (eds) Vulnerabilidad ecológica del golfo de México ante derrames a gran escala. Primera Ed, CIGoM, pp 237–253

[CR5] Améndola-Pimenta M, Cerqueda-García D, Zamora-Briseño JA, Couoh-Puga D, Montero-Muñoz J, Árcega-Cabrera F, Ceja-Moreno V, Pérez-Vega JA, García-Maldonado JQ, del Río-García M, Zapata-Pérez O, Rodríguez-Canul R (2020) Toxicity evaluation and microbiota response of the lined sole *Achirus lineatus* (Chordata: Achiridae) exposed to the light petroleum water-accommodated fraction (WAF). J Toxicol Environ Health - Part A: Curr Issues 83:313–329. 10.1080/15287394.2020.1758861 10.1080/15287394.2020.1758861 32378477

[CR6] Aramaki T, Blanc-Mathieu R, Endo H, Ohkubo K, Kanehisa M, Goto S, Ogata H (2020) KofamKOALA: KEGG Ortholog assignment based on profile HMM and adaptive score threshold. Bioinformatics 36:2251–2252. 10.1093/bioinformatics/btz859 31742321 10.1093/bioinformatics/btz859 PMC7141845

[CR7] Ashburner M, Ball CA, Blake JA, Botstein D, Butler H, Cherry JM, Davis AP, Dolinski K, Dwight SS, Eppig JT, Harris MA, Hill DP, Issel-Tarver L, Kasarskis A, Lewis S, Matese JC, Richardson JE, Ringwald M, Rubin GM, Sherlock G (2000) Gene Ontology. tool for the unification of biology The Gene Ontology Consortium* 10.1038/75556 PMC3037419 10802651

[CR8] Bailey TL, Johnson J, Grant CE, Noble WS (2015) The MEME Suite. Nucleic Acids Res 43:W39–W49. 10.1093/nar/gkv416 25953851 10.1093/nar/gkv416 PMC4489269

[CR9] Bao Z, Eddy SR (2002) Automated *de novo* identification of repeat sequence families in sequenced genomes. Genome Res 12:1269–1276. 10.1101/gr.88502 12176934 10.1101/gr.88502 PMC186642

[CR10] Benson G (1999) Tandem repeats finder: a program to analyze DNA sequences 10.1093/nar/27.2.573 PMC148217 9862982

[CR12] Brown-Peterson NJ, Krasnec M, Takeshita R, Ryan CN, Griffitt KJ, Lay C, Mayer GD, Bayha KM, Hawkins WE, Lipton I, Morris J, Griffitt RJ (2015) A multiple endpoint analysis of the effects of chronic exposure to sediment contaminated with Deepwater Horizon oil on juvenile Southern flounder and their associated microbiomes. Aquat Toxicol 165:197–209. 10.1016/j.aquatox.2015.06.001 26092636 10.1016/j.aquatox.2015.06.001

[CR11] Brown-Peterson NJ, Krasnec M, Takeshita R, Ryan CN, Griffitt KJ, Lay C, Mayer GD, Bayha KM, Hawkins WE, Lipton I, Morris J, Griffitt RJ (2017) Responses of juvenile southern flounder exposed to Deepwater Horizon oil-contaminated sediments. Environ Toxicol Chem 36:1067–1076. 10.1002/etc.3629 27676139 10.1002/etc.3629

[CR13] Buchfink B, Reuter K, Drost HG (2021) Sensitive protein alignments at tree-of-life scale using DIAMOND. Nat Methods 18:366–368. 10.1038/s41592-021-01101-x 33828273 10.1038/s41592-021-01101-x PMC8026399

[CR14] Camacho C, Coulouris G, Avagyan V, Ma N, Papadopoulos J, Bealer K, Madden TL (2009) BLAST+: Architecture and applications. BMC Bioinformatics 10. 10.1186/1471-2105-10-421 10.1186/1471-2105-10-421 PMC2803857 20003500

[CR15] Cañizares-Martinez MA, Quintanilla-Mena M, Del Rio-Garcia M, Rivas-Reyes I, Patiño-Suárez V, Vidal-Martínez VM, Aguirre-Macedo ML, Puch-Hau C (2021) Acute Exposure to Crude Oil Induces Epigenetic, Transcriptional and Metabolic Changes in Juvenile *Sciaenops ocellatus*. Bull Environ Contam Toxicol. 10.1007/s00128-021-03241-4 33914098 10.1007/s00128-021-03241-4

[CR16] Cañizares-Martínez MA, Quintanilla-Mena MA, Améndola-Pimenta M, Rodríguez-Canul R, Árcega-Cabrera F, Del Río-García M, Ceja-Moreno V, Aguirre-Macedo ML, Puch-Hau CA (2024a) Multiple-Integrated Biomarker Indexes to Assess the Responses of the Flatfish *Achirus lineatus* during Exposure to Light Crude Oil Water Accommodated Fraction. Bull Environ Contam Toxicol 113. 10.1007/s00128-024-03967-x 10.1007/s00128-024-03967-x 39427082

[CR17] Cañizares-Martínez MA, Quintanilla-Mena MA, Árcega-Cabrera F, Ceja-Moreno V, Del Río-García M, Reyes-Solian SG, Rivas-Reyes I, Rivera-Bustamante RF, Puch-Hau CA (2024b) Transcriptional Response of Vitellogenin Gene in Flatfish to Environmental Pollutants from Two Regions of the Gulf of Mexico. Bull Environ Contam Toxicol 112:11. 10.1007/s00128-023-03825-2 10.1007/s00128-023-03825-2 38092994

[CR18] Cantalapiedra CP, Hern̗andez-Plaza A, Letunic I, Bork P, Huerta-Cepas J (2021) eggNOG-mapper v2: Functional Annotation, Orthology Assignments, and Domain Prediction at the Metagenomic Scale. Mol Biol Evol 38:5825–5829. 10.1093/molbev/msab293 34597405 10.1093/molbev/msab293 PMC8662613

[CR19] Cantarel BL, Korf I, Robb SMC, Parra G, Ross E, Moore B, Holt C, Alvarado AS, Yandell M (2008) MAKER: An easy-to-use annotation pipeline designed for emerging model organism genomes. Genome Res 18:188–196. 10.1101/gr.6743907 18025269 10.1101/gr.6743907 PMC2134774

[CR20] Carvalho De Azevedo MF, Oliveira C, Pardo BG, Martínez P, Foresti F (2005) Chromosome banding and 18S rDNA in situ hybridization analysis of seven species of the family Achiridae (Teleostei: Pleuronectiformes). Genetica 125:125–132. 10.1007/s10709-005-4921-7 16247686 10.1007/s10709-005-4921-7

[CR21] Cerqueda-García D, Améndola-Pimenta M, Zamora-Briseño JA, González-Penagos CE, Árcega-Cabrera F, Ceja-Moreno V, Rodríguez-Canul R (2020) Effects of chronic exposure to water accommodated fraction (WAF) of light crude oil on gut microbiota composition of the lined sole (*Achirus lineatus*). Mar Environ Res 161. 10.1016/j.marenvres.2020.105116 10.1016/j.marenvres.2020.105116 32861142

[CR22] Chen C, Wu Y, Li J, Wang X, Zeng Z, Xu J, Liu Y, Feng J, Chen H, He Y, Xia R (2023) TBtools-II: A one for all, all for one bioinformatics platform for biological big-data mining. Mol Plant 16:1733–1742. 10.1016/j.molp.2023.09.010 37740491 10.1016/j.molp.2023.09.010

[CR23] Cheng H, Concepcion GT, Feng X, Zhang H, Li H (2021) Haplotype-resolved *de novo* assembly using phased assembly graphs with hifiasm. Nat Methods 18:170–175. 10.1038/s41592-020-01056-5 33526886 10.1038/s41592-020-01056-5 PMC7961889

[CR24] Chuang PC, Young MB, Dale AW, Miller LG, Herrera-Silveira JA, Paytan A (2017) Methane fluxes from tropical coastal lagoons surrounded by mangroves, Yucatán, Mexico. J Geophys Res Biogeosci 122:1156–1174. 10.1002/2017JG003761

[CR25] Cicconardi F, Milanetti E, Pinheiro de Castro EC, Mazo-Vargas A, Van Belleghem SM, Ruggieri AA, Rastas P, Hanly J, Evans E, Jiggins CD, Owen McMillan W, Papa R, Di Marino D, Martin A, Montgomery SH (2023) Evolutionary dynamics of genome size and content during the adaptive radiation of *Heliconiini* butterflies. Nat Commun 14. 10.1038/s41467-023-41412-5 10.1038/s41467-023-41412-5 PMC10497600 37699868

[CR26] Cohen-Sánchez A, Compa M, Lombardo J, Quetglas-Llabrés MM, Ribas-Taberner M, del M, Jiménez-García M, Tejada S, Sureda A (2025) Physiological Stress Responses Associated with Microplastic Ingestion in the Benthic Flatfish *Bothus podas*. Toxics 13. 10.3390/toxics13070584 10.3390/toxics13070584 PMC12298673 40711029

[CR27] De Bie T, Cristianini N, Demuth JP, Hahn MW (2006) CAFE: A computational tool for the study of gene family evolution. Bioinformatics 22:1269–1271. 10.1093/bioinformatics/btl097 16543274 10.1093/bioinformatics/btl097

[CR28] De Coster W, Rademakers R (2023) NanoPack2: population-scale evaluation of long-read sequencing data. Bioinformatics 39. 10.1093/bioinformatics/btad311 10.1093/bioinformatics/btad311 PMC10196664 37171891

[CR29] de Oliveira EC, Fávaro LF (2010) Reproduction of the flatfish *Achirus lineatus* (pleuronectiformes: Achiridae) in paranaguá bay, state of paraná, a subtropical region of Brazil. Zoologia 27:523–532. 10.1590/S1984-46702010000400004

[CR30] Dierking J, Wafo E, Schembri T, Lagadec V, Nicolas C, Letourneur Y, Harmelin-Vivien M (2009) Spatial patterns in PCBs, pesticides, mercury and cadmium in the common sole in the NW Mediterranean Sea, and a novel use of contaminants as biomarkers. Mar Pollut Bull 58:1605–1614. 10.1016/j.marpolbul.2009.07.008 19692097 10.1016/j.marpolbul.2009.07.008

[CR31] Domazet-Lošo M, Široki T, Šimičević K, Domazet-Lošo T (2024) Macroevolutionary dynamics of gene family gain and loss along multicellular eukaryotic lineages. Nat Commun 15. 10.1038/s41467-024-47017-w 10.1038/s41467-024-47017-w PMC10966110 38531970

[CR32] Eddy SR (2011) Accelerated profile HMM searches. PLoS Comput Biol 7. 10.1371/journal.pcbi.1002195 10.1371/journal.pcbi.1002195 PMC3197634 22039361

[CR33] Eide M, Zhang X, Karlsen OA, Goldstone JV, Stegeman J, Jonassen I, Goksøyr A (2021) The chemical defensome of five model teleost fish. Sci Rep 11. 10.1038/s41598-021-89948-0 10.1038/s41598-021-89948-0 PMC8131381 34006915

[CR34] Eirín-López JM, Rebordinos L, Rooney AP, Rozas J (2012) The Birth-and-Death. Evolution of Multigene Families Revisited 10.1159/000337119 22759819

[CR35] Ellinghaus D, Kurtz S, Willhoeft U (2008) LTRharvest, an efficient and flexible software for *de novo* detection of LTR retrotransposons. BMC Bioinformatics 9. 10.1186/1471-2105-9-18 10.1186/1471-2105-9-18 PMC2253517 18194517

[CR36] Emms DM, Kelly S (2019) OrthoFinder: Phylogenetic orthology inference for comparative genomics. Genome Biol 20. 10.1186/s13059-019-1832-y 10.1186/s13059-019-1832-y PMC6857279 31727128

[CR37] Ewels PA, Peltzer A, Fillinger S, Patel H, Alneberg J, Wilm A, Garcia MU, Di Tommaso P, Nahnsen S (2020) The nf-core framework for community-curated bioinformatics pipelines. Nat Biotechnol 38:276–278. 10.1038/s41587-020-0439-x 32055031 10.1038/s41587-020-0439-x

[CR38] Flynn JM, Hubley R, Goubert C, Rosen J, Clark AG, Feschotte C, Smit AF (2020) RepeatModeler2 for automated genomic discovery of transposable element families. Genome Biol 117:9451–9457. 10.1186/s13059-018-1577-z 10.1073/pnas.1921046117 PMC7196820 32300014

[CR39] Franco ME, Araújo CVM, Cerveny D, Koubová A, Danneels B, Goksøyr A, Bertram MG (2025) The extended chemical defensome: emphasizing mechanisms of defense as key research avenues to tackle priority questions in environmental toxicology. Environ Toxicol Chem 44:3118–3130 40728939 10.1093/etojnl/vgaf190

[CR40] Gao T, Liu K, Liu Q, Wang D (2024) An improved chromosome-level genome assembly and annotation of Echeneis naucrates. Sci Data 11. 10.1038/s41597-024-03309-w 10.1038/s41597-024-03309-w PMC11069562 38704456

[CR41] Grabherr MG, Haas BJ, Yassour M, Levin JZ, Thompson DA, Amit I, Adiconis X, Fan L, Raychowdhury R, Zeng Q, Chen Z, Mauceli E, Hacohen N, Gnirke A, Rhind N, Di Palma F, Birren BW, Nusbaum C, Lindblad-Toh K, Friedman N, Regev A (2011) Full-length transcriptome assembly from RNA-Seq data without a reference genome. Nat Biotechnol 29:644–652. 10.1038/nbt.1883 21572440 10.1038/nbt.1883 PMC3571712

[CR42] Gracian Negrete JM (2012) Estatus taxonómico de *Achirus lineatus* (Linnaeus, 1758) y *Achirus mazatlanus*, vol 1869. Achiridae). CICIMAR-IPN, Pleuronectiformes, Steindachner

[CR43] Grant CE, Bailey TL, Noble WS (2011) FIMO: Scanning for occurrences of a given motif. Bioinformatics 27:1017–1018. 10.1093/bioinformatics/btr064 21330290 10.1093/bioinformatics/btr064 PMC3065696

[CR44] Haas BJ, Delcher AL, Mount SM, Wortman JR, Smith RK, Hannick LI, Maiti R, Ronning CM, Rusch DB, Town CD, Salzberg SL, White O (2003) Improving the Arabidopsis genome annotation using maximal transcript alignment assemblies. Nucleic Acids Res 31(19):5654–5666. 10.1093/nar/gkg770 14500829 10.1093/nar/gkg770 PMC206470

[CR45] Haas BJ, Salzberg SL, Zhu W, Pertea M, Allen JE, Orvis J, White O, Robin CR, Wortman JR (2008) Automated eukaryotic gene structure annotation using EVidenceModeler and the Program to Assemble Spliced Alignments. Genome Biol 9(1). 10.1186/gb-2008-9-1-r7 10.1186/gb-2008-9-1-r7 PMC2395244 18190707

[CR46] Hai T, Wolfgang CD, Marsee DK, Allen AE, Sivaprasad U (1999) ATF3 and Stress Responses. Gene Expr 7(4–6):321–335 PMID: 10440233; PMCID: PMC6174666 10440233 PMC6174666

[CR47] Hardage K, Street J, Herrera-Silveira JA, Oberle FKJ, Paytan A (2022) Late Holocene environmental change in Celestun Lagoon. Yucatan Mexico J Paleolimnol 67:131–162. 10.1007/s10933-021-00227-4

[CR48] He F, Ru X, Wen T (2020) NRF2, a transcription factor for stress response and beyond. Int J Mol Sci 21:1–23 10.3390/ijms21134777 PMC7369905 32640524

[CR49] Hernández-Plaza A, Szklarczyk D, Botas J, Cantalapiedra CP, Giner-Lamia J, Mende DR, Kirsch R, Rattei T, Letunic I, Jensen LJ, Bork P, von Mering C, Huerta-Cepas J (2023) eggNOG 6.0: enabling comparative genomics across 12 535 organisms. Nucleic Acids Res 51:D389–D394. 10.1093/nar/gkac1022 36399505 10.1093/nar/gkac1022 PMC9825578

[CR50] Hotamisligil GS, Davis RJ (2016) Cell signaling and stress responses. Cold Spring Harb Perspect Biol 8. 10.1101/cshperspect.a006072 10.1101/cshperspect.a006072 PMC5046695 27698029

[CR51] Huerta-Cepas J, Szklarczyk D, Heller D, Hernández-Plaza A, Forslund SK, Cook H, Mende DR, Letunic I, Rattei T, Jensen LJ, Von Mering C, Bork P (2019) EggNOG 5.0: A hierarchical, functionally and phylogenetically annotated orthology resource based on 5090 organisms and 2502 viruses. Nucleic Acids Res 47:D309–D314. 10.1093/nar/gky1085 30418610 10.1093/nar/gky1085 PMC6324079

[CR52] Iwasaki W, Fukunaga T, Isagozawa R, Yamada K, Maeda Y, Satoh TP, Sado T, Mabuchi K, Takeshima H, Miya M, Nishida M (2013) Mitofish and mitoannotator: A mitochondrial genome database of fish with an accurate and automatic annotation pipeline. Mol Biol Evol 30:2531–2540. 10.1093/molbev/mst141 23955518 10.1093/molbev/mst141 PMC3808866

[CR53] Jin D, Wang N, Xue Y, Yang Y, Shi K, Wu H, Sheu JJC, Jeong JH, Ban Z, Shen D, Yang L (2025) Pan-cancer analysis of ARNT2 and its oncogenic role in cervical cancer. J Gynecol Oncol 36:e113. 10.3802/jgo.2025.36.e113 40488594 10.3802/jgo.2025.36.e113 PMC12636100

[CR54] Johnston W, Adil S, Cao C, Nipu N, Mennigen JA (2025) Fish models to explore epigenetic determinants of hypoxia-tolerance. Comp Biochem Physiol Mol Integr Physiol 302:111811. 10.1016/j.cbpa.2025.111811 10.1016/j.cbpa.2025.111811 39778711

[CR55] Jung JH, Moon YS, Kim BM, Lee YM, Kim M, Rhee JS (2018) Comparative analysis of distinctive transcriptome profiles with biochemical evidence in bisphenol S- and benzo[a]pyrene-exposed liver tissues of the olive flounder *Paralichthys olivaceus*. PLoS ONE 13. 10.1371/journal.pone.0196425 10.1371/journal.pone.0196425 PMC5929548 29715276

[CR56] Kalyaanamoorthy S, Minh BQ, Wong TKF, Von Haeseler A, Jermiin LS (2017) ModelFinder: Fast model selection for accurate phylogenetic estimates. Nat Methods 14:587–589. 10.1038/nmeth.4285 28481363 10.1038/nmeth.4285 PMC5453245

[CR57] Kanehisa M, Sato Y, Morishima K (2016) BlastKOALA and GhostKOALA: KEGG Tools for Functional Characterization of Genome and Metagenome Sequences. J Mol Biol 428:726–731. 10.1016/j.jmb.2015.11.006 26585406 10.1016/j.jmb.2015.11.006

[CR58] Kobelkowsky A (2000) Sistema urogenital de los lenguados de la familia *Achiridae* (Pisces: Pleuronectiformes) del Golfo de México. Hidrobiológica 10:51–60

[CR59] Korf I (2004) Gene finding in novel genomes. BMC Bioinformatics 5:59. 10.1186/1471-2105-5-59 10.1186/1471-2105-5-59 PMC421630 15144565

[CR60] Kreß JKC, Jessen C, Hufnagel A, Schmitz W, Xavier da Silva TN, Ferreira dos Santos A, Mosteo L, Goding CR, Friedmann Angeli JP, Meierjohann S (2023) The integrated stress response effector ATF4 is an obligatory metabolic activator of NRF2. Cell Rep 42. 10.1016/j.celrep.2023.112724 10.1016/j.celrep.2023.112724 37410595

[CR61] Kuk-Dzul JG, Gold-Bouchot G, Ardisson PL (2012) Benthic infauna variability in relation to environmental factors and organic pollutants in tropical coastal lagoons from the northern Yucatan Peninsula. Mar Pollut Bull 64:2725–2733. 10.1016/j.marpolbul.2012.09.022 23103028 10.1016/j.marpolbul.2012.09.022

[CR62] Kumar S, Suleski M, Craig JM, Kasprowicz AE, Sanderford M, Li M, Stecher G, Hedges SB (2022) TimeTree 5: An Expanded Resource for Species Divergence Times. Mol Biol Evol 39. 10.1093/molbev/msac174 10.1093/molbev/msac174 PMC9400175 35932227

[CR63] Laetsch DR, Blaxter ML (2017) BlobTools: Interrogation of genome assemblies. F1000Res 6:1287. 10.12688/f1000research.12232.1

[CR64] Langmead B, Salzberg SL (2012) Fast gapped-read alignment with Bowtie 2. Nat Methods 9:357–359. 10.1038/nmeth.1923 22388286 10.1038/nmeth.1923 PMC3322381

[CR65] Lawrence MJ, Grayson P, Jeffrey JD, Docker MF, Garroway CJ, Wilson JM, Manzon RG, Wilkie MP, Jeffries KM (2022) Variation in the Transcriptome Response and Detoxification Gene Diversity Drives Pesticide Tolerance in Fishes. Environ Sci Technol 56:12137–12147. 10.1021/acs.est.2c00821 35973096 10.1021/acs.est.2c00821

[CR66] Lee BY, Kim DH, Kim HS, Kim BM, Han J, Lee JS (2018) Identification of 74 cytochrome P450 genes and co-localized cytochrome P450 genes of the CYP2K, CYP5A, and CYP46A subfamilies in the mangrove killifish *Kryptolebias marmoratus*. BMC Genomics 19. 10.1186/s12864-017-4410-2 10.1186/s12864-017-4410-2 PMC5751882 29295707

[CR67] Lee SW, Kim B, Seong JB, Park YH, Lee HJ, Lee DS (2023) ATF6 is a critical regulator of cadmium-mediated apoptosis in spermatocytes. Toxicol Sci 194:167–177. 10.1093/toxsci/kfad055 37261864 10.1093/toxsci/kfad055

[CR68] Lewin HA, Robinson GE, Kress WJ, Baker WJ, Coddington J, Crandall KA, Durbin R, Edwards SV, Forest E, Thomas M, Gilbert P, Goldstein MM, Grigoriev IV, Hackett KJ, Haussler D, Jarvis ED, Johnson WE, Patrinos A, Richards S, Carlos, Castilla-Rubio J, Van Sluys M-A, Soltis PS, Xu X, Yang H, Zhang G (2018) Earth BioGenome Project: Sequencing life for the future of life. Royal Botanic Gardens 115:4325–4333. 10.1073/pnas.1720115115/-/DCSupplemental 10.1073/pnas.1720115115 PMC5924910 29686065

[CR69] Li B, Wang G, Zheng X, Liu M, Yang Y, Ren Y, Zhang Y, Liu Y, He Z, Ren J, Wan H, Cao W, Wang Y, Zhang X, Hou J (2025) Exposure to deltamethrin leads to gill liver damage, oxidative stress, inflammation, and metabolic disorders of Japanese flounder (*Paralichthys olivaceus*). Front Toxicol 7. 10.3389/ftox.2025.1560192 10.3389/ftox.2025.1560192 PMC12041085 40309513

[CR70] Liu D, Hunt M, Tsai IJ (2018) Inferring synteny between genome assemblies: A systematic evaluation. BMC Bioinformatics 19. 10.1186/s12859-018-2026-4 10.1186/s12859-018-2026-4 PMC5791376 29382321

[CR71] Liu K, Liu Q, Qu Y, Gao T (2026) Chromosome-level genome assembly of *Chirolophis japonicus* Herzenstein, 1890 (Stichaeidae, Perciformes). 10.1038/s41597-026-06893-1. Sci Data 13 10.1038/s41597-026-06893-1 PMC13066030 41775751

[CR72] Lü Z, Gong L, Ren Y, Chen Y, Wang Z, Liu L, Li H, Chen X, Li Z, Luo H, Jiang H, Zeng Y, Wang Y, Wang K, Zhang C, Jiang H, Wan W, Qin Y, Zhang J, Zhu L, Shi W, He S, Mao B, Wang W, Kong X, Li Y (2021) Large-scale sequencing of flatfish genomes provides insights into the polyphyletic origin of their specialized body plan. Nat Genet 53:742–751. 10.1038/s41588-021-00836-9 33875864 10.1038/s41588-021-00836-9 PMC8110480

[CR73] Manni M, Berkeley MR, Seppey M, Simão FA, Zdobnov EM (2021a) BUSCO Update: Novel and Streamlined Workflows along with Broader and Deeper Phylogenetic Coverage for Scoring of Eukaryotic, Prokaryotic, and Viral Genomes. Mol Biol Evol 38:4647–4654. 10.1093/molbev/msab199 34320186 10.1093/molbev/msab199 PMC8476166

[CR74] Manni M, Berkeley MR, Seppey M, Zdobnov EM (2021b) BUSCO: assessing genomic data quality and beyond. Curr Protoc 1. 10.1002/cpz1.323 10.1002/cpz1.323 34936221

[CR75] Marcuzzo C, Birbes C, Eché C, Di Franco A, Faraut T, Denis E, Kuchly C, Vernette C, Praud S, Charcosset A, Gaspin C, Milan D, Nicolas SD, Donnadieu C, Vitte C, Klopp C, Iampietro C (2026) High-quality chromosome-scale genome assemblies of 29 maize inbred lines of European breeding relevance. Sci Data 13. 10.1038/s41597-026-07055-z 10.1038/s41597-026-07055-z PMC13153409 41857058

[CR76] Mendes FK, Vanderpool D, Fulton B, Hahn MW (2020) CAFE 5 models variation in evolutionary rates among gene families. Bioinformatics 36:5516–5518. 10.1093/bioinformatics/btaa1022 10.1093/bioinformatics/btaa1022 33325502

[CR77] Mikheenko A, Saveliev V, Hirsch P, Gurevich A (2023) WebQUAST: Online evaluation of genome assemblies. Nucleic Acids Res 51:W601–W606. 10.1093/nar/gkad406 37194696 10.1093/nar/gkad406 PMC10320133

[CR78] Mirbahai L, Southam AD, Sommer U, Williams TD, Bignell JP, Lyons BP, Viant MR, Chipman JK (2013) Disruption of DNA methylation via S-adenosylhomocysteine is a key process in high incidence liver carcinogenesis in fish. J Proteome Res 12:2895–2904. 10.1021/pr400195u 23611792 10.1021/pr400195u

[CR79] Mwamburi SM, Kawato S, Furukawa M, Konishi K, Nozaki R, Hirono I, Kondo H (2024) *De novo* assembly and annotation of the siganus fuscescens (Houttuyn, 1782) genome: marking a pioneering advance for the *siganidae* family. Mar Biotechnol 26:902–916. 10.1007/s10126-024-10325-9 10.1007/s10126-024-10325-9 38850360

[CR80] Nevers Y, Warwick Vesztrocy A, Rossier V, Train CM, Altenhoff A, Dessimoz C, Glover NM (2025) Quality assessment of gene repertoire annotations with OMArk. Nat Biotechnol 43:124–133. 10.1038/s41587-024-02147-w 38383603 10.1038/s41587-024-02147-w PMC11738984

[CR81] Nguyen LT, Schmidt HA, Von Haeseler A, Minh BQ (2015) IQ-TREE: A fast and effective stochastic algorithm for estimating maximum-likelihood phylogenies. Mol Biol Evol 32:268–274. 10.1093/molbev/msu300 25371430 10.1093/molbev/msu300 PMC4271533

[CR82] Olson MV (1999) When Less Is More: Gene Loss as an Engine of Evolutionary Change. Am J Hum Genet 64:18–23 9915938 10.1086/302219 PMC1377697

[CR83] Ou S, Jiang N (2018) LTR_retriever: A highly accurate and sensitive program for identification of long terminal repeat retrotransposons. Plant Physiol 176:1410–1422. 10.1104/pp.17.01310 29233850 10.1104/pp.17.01310 PMC5813529

[CR84] Partheeban EC, Anbazhagan V, Arumugam G, Seshasayanan B, Rajendran R, Paray BA, Al-Sadoon MK, Al-Mfarij AR (2021) Evaluation of toxic metal contaminants in the demersal flatfishes (Order: Pleuronectiformes) collected from a marine biosphere reserve. Reg Stud Mar Sci 42. 10.1016/j.rsma.2021.101649

[CR85] PEMEX (2026) Instruye presidenta Claudia Sheinbaum investigación; separan a 3 funcionarios de cargo por derrame en Golfo de México. Available at: https://www.pemex.com/saladeprensa/boletines_nacionales/Paginas/2026_43_nacional.aspx (Accessed April 2026)

[CR86] Price AL, Jones NC, Pevzner PA (2005) *De novo* identification of repeat families in large genomes. Bioinformatics 21:i351–i358. 10.1093/bioinformatics/bti1018 15961478 10.1093/bioinformatics/bti1018

[CR87] Puch-Hau C, Zapata-Pérez O, Rivas-Reyes I, Quintanilla-Mena M, Del Rio-Garcia M, Gonzalez-Rivera L (2016) Analysis of gene expression biomarkers associated with the presence of pollutans in *Syacium guntery* and *Ariopsis felis* in the Gulf of Mexico. Toxicol Lett 259:S104

[CR88] Pulster EL, Gracia A, Armenteros M, Toro-Farmer G, Snyder SM, Carr BE, Schwaab MR, Nicholson TJ, Mrowicki J, Murawski SA (2020) A First Comprehensive Baseline of Hydrocarbon Pollution in Gulf of Mexico Fishes. Sci Rep 10. 10.1038/s41598-020-62944-6 10.1038/s41598-020-62944-6 PMC7160155 32296072

[CR89] Quintanilla-Mena M, Gold-Bouchot G, Zapata-Pérez O, Rubio-Piña J, Quiroz-Moreno A, Vidal-Martínez VM, Aguirre-Macedo ML, Puch-Hau C (2020) Biological responses of shoal flounder (*Syacium gunteri*) to toxic environmental pollutants from the southern Gulf of Mexico. Environ Pollut 258:10. 10.1016/j.envpol.2019.113669 10.1016/j.envpol.2019.113669 31806456

[CR90] Ranallo-Benavidez TR, Jaron KS, Schatz MC (2020) GenomeScope 2.0 and Smudgeplot for reference-free profiling of polyploid genomes. Nat Commun 11. 10.1038/s41467-020-14998-3 10.1038/s41467-020-14998-3 PMC7080791 32188846

[CR91] Reinar WB, Tørresen OK, Nederbragt AJ, Matschiner M, Jentoft S, Jakobsen KS (2023) Teleost genomic repeat landscapes in light of diversification rates and ecology. Mob DNA 14. 10.1186/s13100-023-00302-9 10.1186/s13100-023-00302-9 PMC10546739 37789366

[CR92] Rhie A, Walenz BP, Koren S, Phillippy AM (2020) Merqury: Reference-free quality, completeness, and phasing assessment for genome assemblies. Genome Biol 21. 10.1186/s13059-020-02134-9 10.1186/s13059-020-02134-9 PMC7488777 32928274

[CR93] Robins CR, Ray GC, Douglass J (1986) A field guide to Atlantic coast fishes of North America. Houghton Mifflin Company, Boston

[CR94] Rocha MLF, Dias JF, Boufleur LA, Santos CEI, Dias JF (2014) Metal concentration in muscle of two species of flatfish from Santos Bay, Southeastern Brazilian coast. Nucl Instrum Methods Phys Res B 318:88–93. 10.1016/j.nimb.2013.05.107

[CR95] Roubeix V, Wessel N, Akcha F, Aminot Y, Briaudeau T, Burgeot T, Chouvelon T, Izagirre U, Munschy C, Mauffret A (2023) Differences in biomarker responses and chemical contamination among three flatfish species in the Bay of Seine (NE Atlantic). Mar Pollut Bull 197. 10.1016/j.marpolbul.2023.115674 10.1016/j.marpolbul.2023.115674 39491290

[CR96] Saary P, Mitchell AL, Finn RD (2020) Estimating the quality of eukaryotic genomes recovered from metagenomic analysis with EukCC. Genome Biol 21. 10.1186/s13059-020-02155-4 10.1186/s13059-020-02155-4 PMC7488429 32912302

[CR97] Sasaki S, Urabe M, Maeda T, Suzuki J, Irie R, Suzuki M, Tomaru Y, Sakaguchi M, Gonzalez FJ, Inoue Y (2018) Induction of Hepatic Metabolic Functions by a Novel Variant of Hepatocyte Nuclear Factor 4. Mol Cell Biol 38. 10.1128/MCB 10.1128/MCB.00213-18 PMC6275183 30224520

[CR98] Sato Y, Miya M, Fukunaga T, Sado T, Iwasaki W (2018) MitoFish and mifish pipeline: A mitochondrial genome database of fish with an analysis pipeline for environmental DNA metabarcoding. Mol Biol Evol 35:1553–1555. 10.1093/molbev/msy074 29668970 10.1093/molbev/msy074 PMC5967551

[CR99] Sepp T, Baines C, Kreitsberg R, Scharsack JP, Nogueira P, Lang T, Fort J, Sild E, Clarke JT, Tuvikene A, Meitern R (2024) Differences on the level of hepatic transcriptome between two flatfish species in response to liver cancer and environmental pollution levels. 275. Comparative Biochemistry and Physiology Part - C: Toxicology and Pharmacology10.1016/j.cbpc.2023.109781 10.1016/j.cbpc.2023.109781 37923151

[CR100] Shahriar S, Patel TD, Nakka M, Grimm SL, Coarfa C, Gorelick DA (2025) Functional genomic analysis of non-canonical DNA regulatory elements of the aryl hydrocarbon receptor. Toxicol Sci. 10.1093/toxsci/kfaf146 10.1093/toxsci/kfaf146 PMC12863213 41128614

[CR101] Shi W, Chen S, Kong X, Si L, Gong L, Zhang Y, Yu H (2018) Flatfish monophyly refereed by the relationship of Psettodes in Carangimorphariae. BMC Genomics 19. 10.1186/s12864-018-4788-5 10.1186/s12864-018-4788-5 PMC5970519 29801430

[CR102] Simão FA, Waterhouse RM, Ioannidis P, Kriventseva EV, Zdobnov EM (2015) BUSCO: Assessing genome assembly and annotation completeness with single-copy orthologs. Bioinformatics 31:3210–3212. 10.1093/bioinformatics/btv351 26059717 10.1093/bioinformatics/btv351

[CR103] Smit AFA, Hubley R, Green P (2015) RepeatMasker Open-4.0. 2013–2015. http://www.repeatmasker.org

[CR104] Smith JJ, Kuraku S, Holt C, Sauka-Spengler T, Jiang N, Campbell MS, Yandell MD, Manousaki T, Meyer A, Bloom OE, Morgan JR, Buxbaum JD, Sachidanandam R, Sims C, Garruss AS, Cook M, Krumlauf R, Wiedemann LM, Sower SA, Decatur WA, Hall JA, Amemiya CT, Saha NR, Buckley KM, Rast JP, Das S, Hirano M, McCurley N, Guo P, Rohner N, Tabin CJ, Piccinelli P, Elgar G, Ruffier M, Aken BL, Searle SMJ, Muffato M, Pignatelli M, Herrero J, Jones M, Brown CT, Chung-Davidson YW, Nanlohy KG, Libants SV, Yeh CY, McCauley DW, Langeland JA, Pancer Z, Fritzsch B, De Jong PJ, Zhu B, Fulton LL, Theising B, Flicek P, Bronner ME, Warren WC, Clifton SW, Wilson RK, Li W (2013) Sequencing of the sea lamprey (*Petromyzon marinus*) genome provides insights into vertebrate evolution. Nat Genet 45:415–421. 10.1038/ng.2568 23435085 10.1038/ng.2568 PMC3709584

[CR105] Song JY, Nakayama K, Kokushi E, Ito K, Uno S, Koyama J, Rahman MH, Murakami Y, Kitamura SI (2012) Effect of heavy oil exposure on antibacterial activity and expression of immune-related genes in Japanese flounder *Paralichthys olivaceus*. Environ Toxicol Chem 31:828–835. 10.1002/etc.1743 22228536 10.1002/etc.1743

[CR106] Stanke M, Diekhans M, Baertsch R, Haussler D (2008) Using native and syntenically mapped cDNA alignments to improve *de novo* gene finding. Bioinformatics 24:637–644. 10.1093/bioinformatics/btn013 18218656 10.1093/bioinformatics/btn013

[CR107] Sun J, Lu F, Luo Y, Bie L, Xu L, Wang Y (2023) OrthoVenn3: An integrated platform for exploring and visualizing orthologous data across genomes. Nucleic Acids Res 51:W397–W403. 10.1093/nar/gkad313 37114999 10.1093/nar/gkad313 PMC10320085

[CR108] Sun Z, Li S, Liu Y, Li W, Liu K, Cao X, Lin J, Wang H, Wang Q, Shao C (2024) Telomere-to-telomere gapless genome assembly of the Chinese sea bass (*Lateolabrax maculatus*). Sci Data 11. 10.1038/s41597-024-02988-9 10.1038/s41597-024-02988-9 PMC10850130 38326339

[CR109] Suzek BE, Wang Y, Huang H, McGarvey PB, Wu CH (2014) UniRef clusters: A comprehensive and scalable alternative for improving sequence similarity searches. Bioinformatics 31:926–932. 10.1093/bioinformatics/btu739 25398609 10.1093/bioinformatics/btu739 PMC4375400

[CR110] Thi Hoang D, Chernomor O, von Haeseler A, Quang Minh B, Sy Vinh L, Rosenberg MS (2017) UFBoot2: Improving the Ultrafast Bootstrap Approximation. Mol Biol Evol 35:518–522. 10.5281/zenodo.854445 10.1093/molbev/msx281 PMC5850222 29077904

[CR111] Uliano-Silva M, Ferreira JGRN, Krasheninnikova K, Blaxter M, Mieszkowska N, Hall N, Holland P, Durbin R, Richards T, Kersey P, Hollingsworth P, Wilson W, Twyford A, Gaya E, Lawniczak M, Lewis O, Broad G, Martin F, Hart M, Barnes I, Formenti G, Abueg L, Torrance J, Myers EW, Durbin R, Blaxter M, McCarthy SA (2023) MitoHiFi: a python pipeline for mitochondrial genome assembly from PacBio high fidelity reads. BMC Bioinformatics 24. 10.1186/s12859-023-05385-y 10.1186/s12859-023-05385-y PMC10354987 37464285

[CR112] Valencia-Pesqueira LM, Hoff SNK, Tørresen OK, Jentoft S, Lefevre S (2025) Chromosome-level *de novo* genome assembly of wild, anoxia-tolerant crucian carp, *Carassius carassius*. Sci Data 12. 10.1038/s41597-025-04813-3 10.1038/s41597-025-04813-3 PMC11933416 40128231

[CR114] Wang R, Song N, Zhao L (2025) Chromosome-Level Genome Assembly and Comparative Genomic Analysis of *Planiliza haematocheilus*: Insights into Environmental Adaptation and Hypoxia Tolerance Mechanisms. Marine Biotech 27. 10.1007/s10126-025-10419-y 10.1007/s10126-025-10419-y 39878786

[CR113] Wang P, Wang X, Yin D, Liu J, Jiang M, Liu K (2026a) Telomere-to-telomere gap-free genome assembly of the *Opsariichthys evolans* (Cypriniformes: Cyprinidae). Sci Data 13. 10.1038/s41597-026-06588-7 10.1038/s41597-026-06588-7 PMC12917274 41571710

[CR115] Wang X, Huang Q, Qin Z, Fan D, Ren C, Pan W, Huang J, Zhang Z, Ge H, Liang J, Xu J, Zhang Y, Luo P, Jiang X, Sun L, Sun H, Hu C, Yan A, Chen T (2026b) Chromosome-level genome assembly and annotation of the tropical sea cucumber *Holothuria fuscocinerea*. Sci Data 13:281. 10.1038/s41597-026-06609-5 41580434 10.1038/s41597-026-06609-5 PMC12920896

[CR116] Wu Y, Wang Y, Wang M, Sun N, Li C (2017) GRIN2A polymorphisms and expression levels are associated with lead-induced neurotoxicity. Toxicol Ind Health 33:332–339. 10.1177/0748233716647636 27230353 10.1177/0748233716647636

[CR117] Wyatt C (2026) Gene EXpansion and CONtraction analysis pipeline. WorkflowHub. 10.48546/WORKFLOWHUB.WORKFLOW.2141.8

[CR118] Xu Xwen, Zheng W, Yang Y, Hou J, Chen S (2022) High-quality Japanese flounder genome aids in identifying stress-related genes using gene coexpression network. Sci Data 9. 10.1038/s41597-022-01821-5 10.1038/s41597-022-01821-5 PMC9668919 36385241

[CR119] Yan T, Gao K, He L, Pu Y, Tang Z, Xiong J, Chen Q, Lai B, Liu F, Chen P, Chen M, Luo W, Huang J, Ding W, Yang D, He Z (2025) Genomic-based revelation of genetic structure and adaptive characterization of *Schizopygopsis malacanthus* in the Jinsha River and Yalong River. BMC Genomics 26. 10.1186/s12864-025-12065-z 10.1186/s12864-025-12065-z PMC12482372 41023589

[CR120] Yasmin F, Marwick KFM, Hunter DW, Nawaz S, Marshall GF, Booker SA, Hardingham GE, Kind PC, Wyllie DJA (2025) Absence of GluN2A in hippocampal CA1 neurons leads to altered dendritic structure and reduced frequency of miniature excitatory synaptic events. Brain Commun 7. 10.1093/braincomms/fcaf124 10.1093/braincomms/fcaf124 PMC11986202 40226380

[CR121] Zamora-Briseño AJ, Améndola-Pimenta M, Antonio Ortega-Rosas D, Pereira-Santana A, Hernández-Velázquez IM, González-Penagos CE, Pérez-Vega JA, del Río-García M, Árcega-Cabrera F, Rodríguez-Canul R (2021) Gill and liver transcriptomic responses of *Achirus lineatus* (Neopterygii: Achiridae) exposed to water-accommodated fraction (WAF) of light crude oil reveal an onset of hypoxia-like condition. Environ Sci Pollut Res 28. 10.1007/s11356-021-12909-7/Published 10.1007/s11356-021-12909-7 33646544

[CR123] Zhang H, Xu J, Long Y, Maimaitijiang A, Su Z, Li W, Li J (2024) Unraveling the guardian: p53’s multifaceted role in the DNA damage response and tumor treatment strategies. Int J Mol Sci 25:12928. 10.3390/ijms252312928 10.3390/ijms252312928 PMC11641486 39684639

[CR122] Zhang D, Hu Q, He T, Zhou J, Wen Y, Liu Q, Zhang J, Zhi W, Ouyang L, Gao S, Guan R, Zhou Z (2026) Assembling a chromosome-level genome for the *Microtus fortis* using PacBio HiFi and Hi-C technologies. Sci Data 13. 10.1038/s41597-026-06813-3 10.1038/s41597-026-06813-3 PMC13018634 41688456

[CR124] Zhou C, Liu Q, Qu Y, Qiao Y, Gao T, Wang D (2024) The first chromosomal-level genome assembly and annotation of white suckerfish *Remora albescens*. Sci Data 11. 10.1038/s41597-024-03363-4 10.1038/s41597-024-03363-4 PMC11111791 38778061

[CR125] Zhu L, Qu K, Xia B, Sun X, Chen B (2016) Transcriptomic response to water accommodated fraction of crude oil exposure in the gill of Japanese flounder, *Paralichthys olivaceus*. Mar Pollut Bull 106:283–291. 10.1016/j.marpolbul.2015.12.022 27001715 10.1016/j.marpolbul.2015.12.022

[CR126] Zhu T, Sato Y, Sado T, Miya M, Iwasaki W (2023) MitoFish, MitoAnnotator, and MiFish Pipeline: Updates in 10 Years. Mol Biol Evol 40. 10.1093/molbev/msad035 10.1093/molbev/msad035 PMC9989731 36857197

