# Supplementary material for: De novo Genome Assembly, Annotation, and Comparative Analysis of the Lined Sole Achirus lineatus as a Resource for Evolutionary and Environmental Genomics
Source: Mar Biotechnol (NY). 2026 Jun 30;28(4):112. doi: 10.1007/s10126-026-10665-8 (PMC13319265; doi:10.1007/s10126-026-10665-8)
Supplement: Supplementary file 6 — Supplementary Material 6 (DOCX 1 MB) [file 10126_2026_10665_MOESM6_ESM.docx]

***De novo* genome assembly, annotation, and comparative analysis of the lined sole *Achirus lineatus* as a resource for evolutionary and environmental genomics**

Mercedes Quintanilla-Mena ^1^ (ORCID: [0000-0003-2835-6087](https://orcid.org/0000-0003-2835-6087)), Elsa B. Góngora-Castillo ^1, 2^ (ORCID: [0000-0001-6327-6993](https://orcid.org/0000-0001-6327-6993)), Rossanna Rodriguez-Canul ^1^ (ORCID: [0000-0003-0469-1489](https://orcid.org/0000-0003-0469-1489)), Rafael Rivera-Bustamante ^1^ (ORCID: [0000-0002-5114-2370](https://orcid.org/0000-0002-5114-2370))

^1^ Departamento de Recursos del Mar, Cinvestav Unidad Mérida, Km 6 Carretera Antigua a Progreso, Mérida, Yucatán, México 97310.

^2^ Secihti-Departamento de Recursos del Mar, Cinvestav Unidad Mérida, Km 6 Carretera Antigua a Progreso, Mérida, Yucatán, México 97310

*Corresponding author: Dr. Rafael Rivera-Bustamante/Dra. Rossanna Rodríguez-Canul. Email: [rriverab@cinvestav.mx](mailto:rriverab@cinvestav.mx); [rossana.rodriguez@cinvestav.mx](mailto:rossana.rodriguez@cinvestav.mx)

**Supplementary Table 1** Software packages and pipelines used for assembly and annotation

| \| Step \| Description \| Software \| Version \| \| --- \| --- \| --- \| --- \| \| Preprocessing \| Raw adapter verification \| HiFiAdapterFilt \| 1.0.0 \| \| Quality verification \| NanoPlot \| 1.46.1 \| \| Fastq filtering \| chopper \| 0.8.0 \| \| Assembly \| K-mer counts \| Meryl \| 1.4 \| \| K-mer quality control and genomic profile \| GenomeScope2 (web) \| 2024 \| \| *de novo* genome assembly \| Hifiasm \| 0.19.9-r616 \| \| *de novo* mitogenome assembly and annotation \| MitoHiFi \| 3.2.3+galaxy0 \| \| Mitogenome visualization \| Mitofish (MitoDraw) \| 2025.06 \| \| Quality control of the assembly \| Continuity metrics \| QUAST \| 5.2.0 \| \| Completeness validation (actinopterygii_odb10) \| BUSCO \| 5.7.0 \| \| Assembly quality value \| Merqury \| 1.3 \| \| Reads mapped to the assembly \| pbmm2 \| 1.14.99 \| \| Cross-contamination verification \| BlobTools \| 1.1.1 \| \| Repetitive genome annotation \| de novo modeling \| RepeatModeler \| 2.0.5 \| \| LTR Structural Analysis \| LTR_Harvest/LTR_Retriever \| 1.6.2/2.9.0 \| \| Detection of tandem repeats \| TandemRepeatsFinder \| 4.09 \| \| Detection of frequently occurring repeats \| RepeatScout \| 1.0.6 \| \| Detection of complex repetitive elements \| RECON \| 1.08 \| \| Detection of repetitive elements; soft -masking; hard-masking \| RepeatMasker (Dfam v38) \| 4.1.7-p \| \| Structural annotation \| Integration of the structural annotation \| MAKER \| 2.31.11 \| \| *ab initio* prediction \| Augustus \| 3.5.0 \| \| *ab initio* prediction \| SNAP \| 2.68.5 \| \| Homologous alignments against other flatfish \| tblastn tool \| 2.9.0 \| \| Alignments of structural predictions for genes, introns, exons, UTRs, etc. \| Exonerate \| 2.4.0 \| \| Reference transcriptome assembly (PRJNA646280) for transcriptomic evidence \| Trinity \| 2.15.1 \| \| Reference transcriptome assembly evaluation \| Bowti2 \| 2.4.5 \| \| Annotation refinement \| PASA \| 2.5.3 \| \| Functional annotation \| Search against UniProt-UniRef90 \| BLASTp \| 2.9.0+ \| \| Search against multiple database \| Eggnog-mapper/Diamond \| 2.1.12/2.1.11 \| \| Search against KOfam HMM ortholog database \| KofamKOALA (HMMER) \| 2025 \| \| Search against KEGG ortholog database \| BlastKOALA \| 3.1 \| \| Comparative genomics \| Quality control of the annotation (predicted proteome) \| BUSCO/OMArk \| 5.7.0/0.3.1 \| \| Scaffolding \| ragtag \| 2.1.0 \| \| Orthology evaluation \| OrthoFinder \| 3.1.0 \| \| Orthology visualization \| OrthoVenn3 (web) \| 3 \| \| Phylogenetic construction \| IQ-TREE \| 1.6.12 \| \| Expansion/contraction analyses \| EXCON \| 2.3.1 \| \|  \| Detection and annotation of cis-regulatory elements (CREs) \| FIMO/MEME Suite \| 5.5.8 \| \|  \| Motif enrichment \| FIMO/MEME Suite \| 5.5.9 web \| |
| --- | --- | --- | --- | --- | --- | --- | --- | --- | --- | --- | --- | --- | --- | --- | --- | --- | --- | --- | --- | --- | --- | --- | --- | --- | --- | --- | --- | --- | --- | --- | --- | --- | --- | --- | --- | --- | --- | --- | --- | --- | --- | --- | --- | --- | --- | --- | --- | --- | --- | --- | --- | --- | --- | --- | --- | --- | --- | --- | --- | --- | --- | --- | --- | --- | --- | --- | --- | --- | --- | --- | --- | --- | --- | --- | --- | --- | --- | --- | --- | --- | --- | --- | --- | --- | --- | --- | --- | --- | --- | --- | --- | --- | --- | --- | --- | --- | --- | --- | --- | --- | --- | --- | --- | --- | --- | --- | --- | --- | --- | --- | --- | --- | --- | --- | --- | --- | --- | --- | --- | --- | --- | --- | --- | --- | --- | --- | --- | --- | --- | --- |

**Supplementary Table 2** Statistics on transposable elements in the *Achiurus lineatus* genome

| \| Type \| Length (Bp) \| % in genome \| \| --- \| --- \| --- \| \| DNA \| 12,928,846 \| 2.66 \| \| LINE \| 143,59,474 \| 2.95 \| \| SINE \| 956,256 \| 0.2 \| \| LTR \| 28,300,051 \| 5.82 \| \| Satellite \| 0 \| 0 \| \| Simple_repeat \| 18,080,579 \| 3.72 \| \| Other \| 4862502 \| 1 \| \| Unknown \| 52,322,094 \| 10.76 \| \| Total \| 117,450,328 \| 27.11 \| |
| --- | --- | --- | --- | --- | --- | --- | --- | --- | --- | --- | --- | --- | --- | --- | --- | --- | --- | --- | --- | --- | --- | --- | --- | --- | --- | --- | --- | --- | --- | --- |

**Supplementary Table 3** Final gene models produced by MAKER and annotation metrics of the protein-coding genes in *Achirus lineatus* genome

| Metric | | | | Value | |
| --- | --- | --- | --- | --- | --- |
| Number of gene predicted | | | | 22,412 | |
| Number of exon | | | | 246,727 | |
| Retained *ab initio* predictions (AUGUSTUS/SNAP) | | | | 3,029 | |
| Number of genes supported only by transcript evidence | | | | 640 (2.86%) | |
| Number of genes supported only by protein evidence | | | | 5,577 (24.88%) | |
| Number of genes supported by both transcript and protein evidence | | | | 16,195 (72.26%) | |
| Number of mrna | | | | 22,412 | |
| Number of mrnas with utr both sides | | | | 6,346 | |
| Number of mrnas with at least one utr | | | | 12,036 | |
| Number of cds | | | | 22,412 | |
| Number of five_prime_utr | | | | 8,818 | |
| Number of three_prime_utr | | | | 9,564 | |
| mean exons per mrna | | | | 11 | |
| mean exons per cds | | | | 10.7 | |
| mean introns in cdss per mrna | | | | 9.7 | |
| mean introns in exons per mrna | | | | 10 | |
| Total gene length (bp) | | | | 208,121,475 | |
| Total mrna length (bp) | | | | 208,121,475 | |
| Total cds length (bp) | | | | 39,791,013 | |
| Total exon length (bp) | | | | 50,012,459 | |
| mean gene length (bp) | | | | 9,286 | |
| mean mrna length (bp) | | | | 9,286 | |
| mean cds length (bp) | | | | 1,775 | |
| mean exon length (bp) | | | | 203 | |
| mean five_prime_utr length (bp) | | | | 225 | |
| mean three_prime_utr length (bp) | | | | 861 | |
| **Supplementary Table 4** Comparison of structural annotation metrics among gene prediction pipelines. | | | | |  |
| Feature | Helixer *ab initio* deep learning | MAKER | MAKER+PASA (longest isoform) | |  |
| Number of genes | 24,415 | 22,412 | 22,100 | |  |
| Number of mRNAs | 24,415 | 22,412 | 22,100 | |  |
| Number of CDSs | 24,415 | 22,412 | 22,100 | |  |
| Number of exons | 287,140 | 246,727 | 243,659 | |  |
| Mean gene length (bp) | 12,456 | 9,286 | 10,539 | |  |
| Mean CDS length (bp) | 1,708 | 1,775 | 1,780 | |  |
| Mean exon length (bp) | 243 | 203 | 224 | |  |
| Mean exons per mRNA | 12 | 11 | 11 | |  |
| Monoexonic genes | 675 | 320 | 264 | |  |
| Multiexonic genes | 23,740 | 22,092 | 21,836 | |  |
| Shared gene models with MAKER | 21,273 |  | 21,808 | |  |

**Supplementary Table 5** Summary of functional annotations for predicted genes of *Achirus lineatus* genome

|  |  | Number | Percent (%) |
| --- | --- | --- | --- |
| Total |  | 22412 | 100 |
| Aannotated | UniProt-UniRef90 | 21469 | 95.79 |
|  | Emapper (eggNOG) | 20223 | 90.23 |
|  | EggNog-Pfam | 19544 | 87.20 |
|  | EggNog-KEGG | 15043 | 67.12 |
|  | EggNog-GO | 14062 | 62.74 |
|  | KEGG (BlastKOALA) | 13608 | 60.72 |
|  | KOfam (KofamKOALA) | 13805 | 61.60 |
|  | Total | 21503 | 95.94 |
| Unannotated |  | 909 | 4.06 |

**Supplementary Table 6** Summary of orthogroups inferred by OrthoFinder

|  |  | Value |
| --- | --- | --- |
| Number of species |  | 8 |
| Number of genes |  | 198057 |
| Number of genes in orthogroups |  | 191294 |
| Number of unassigned genes |  | 6763 |
| Percentage of genes in orthogroups |  | 96.6 |
| Percentage of unassigned genes |  | 3.4 |
| Number of orthogroups |  | 20760 |
| Number of species-specific orthogroups |  | 751 |
| Number of genes in species-specific orthogroups |  | 3606 |
| Percentage of genes in species-specific orthogroups |  | 1.8 |
| Mean orthogroup size |  | 9.2 |
| Median orthogroup size |  | 8 |
| G50 (assigned genes) |  | 9 |
| G50 (all genes) |  | 9 |
| O50 (assigned genes) |  | 7304 |
| O50 (all genes) |  | 7679 |
| Number of orthogroups with all species present |  | 12449 |
| Number of single-copy orthogroups |  | 5840 |

| **Supplementary Table 7.** Comparison of predicted protein-coding genes (longest isoform per gene) and corresponding KEGG annotations between *A. lineatus* and other flatfish species. | | |
| --- | --- | --- |
| Species | Protein-coding genes (longest isoform set) | KEGG-annotated genes |
| *Achirus lineatus* | 22100 | 15043 |
| *Paralichthys olivaceus* | 22134 | 18455 |
| *Cynoglossus semilaevis* | 21420 | 15714 |
| *Hippoglossus hippoglossus* | 22234 | 18320 |
| *Hippoglossus stenolepis* | 21933 | 17501 |
| *Solea solea* | 22383 | 17876 |

**Supplementary Table 8.** Comparative counts of KEGG-annotated genes, genes associated with xenobiotic biodegradation and metabolism pathways, and major Phase I and Phase II xenobiotic biotransformation gene families across species

| \|  \| \| \| \| \| \| \| \| \| \| --- \| --- \| --- \| --- \| --- \| --- \| --- \| --- \| --- \| \| Species \| KEGG-annotated genes \| Genes associated with xenobiotic biodegradation and metabolism pathways \| CYP450 Phase I \| Hydrolase Phase I \| Redox enzyme Phase I \| GST Phase II \| UGT Phase II \| Other xenobiotic related \| \| *Achirus lineatus* \| 15043 \| 123 \| 8 \| 5 \| 38 \| 10 \| 11 \| 51 \| \| *Paralichthys olivaceus* \| 18455 \| 162 \| 11 \| 5 \| 55 \| 15 \| 13 \| 63 \| \| *Cynoglossus semilaevis* \| 15714 \| 144 \| 7 \| 3 \| 43 \| 10 \| 22 \| 59 \| \| *Hippoglossus hippoglossus* \| 18320 \| 168 \| 11 \| 4 \| 53 \| 17 \| 15 \| 68 \| \| *Hippoglossus stenolepis* \| 17501 \| 168 \| 13 \| 11 \| 47 \| 18 \| 17 \| 62 \| \| *Platichthys flesus* \| 17381 \| 157 \| 11 \| 4 \| 44 \| 17 \| 17 \| 64 \| \| *Solea solea* \| 17876 \| 177 \| 12 \| 4 \| 47 \| 16 \| 25 \| 73 \| \| *Danio rerio* \| 21892 \| 235 \| 9 \| 11 \| 59 \| 33 \| 39 \| 84 \| |
| --- | --- | --- | --- | --- | --- | --- | --- | --- | --- | --- | --- | --- | --- | --- | --- | --- | --- | --- | --- | --- | --- | --- | --- | --- | --- | --- | --- | --- | --- | --- | --- | --- | --- | --- | --- | --- | --- | --- | --- | --- | --- | --- | --- | --- | --- | --- | --- | --- | --- | --- | --- | --- | --- | --- | --- | --- | --- | --- | --- | --- | --- | --- | --- | --- | --- | --- | --- | --- | --- | --- | --- | --- | --- | --- | --- | --- | --- | --- | --- | --- | --- | --- | --- | --- | --- | --- | --- | --- | --- | --- |


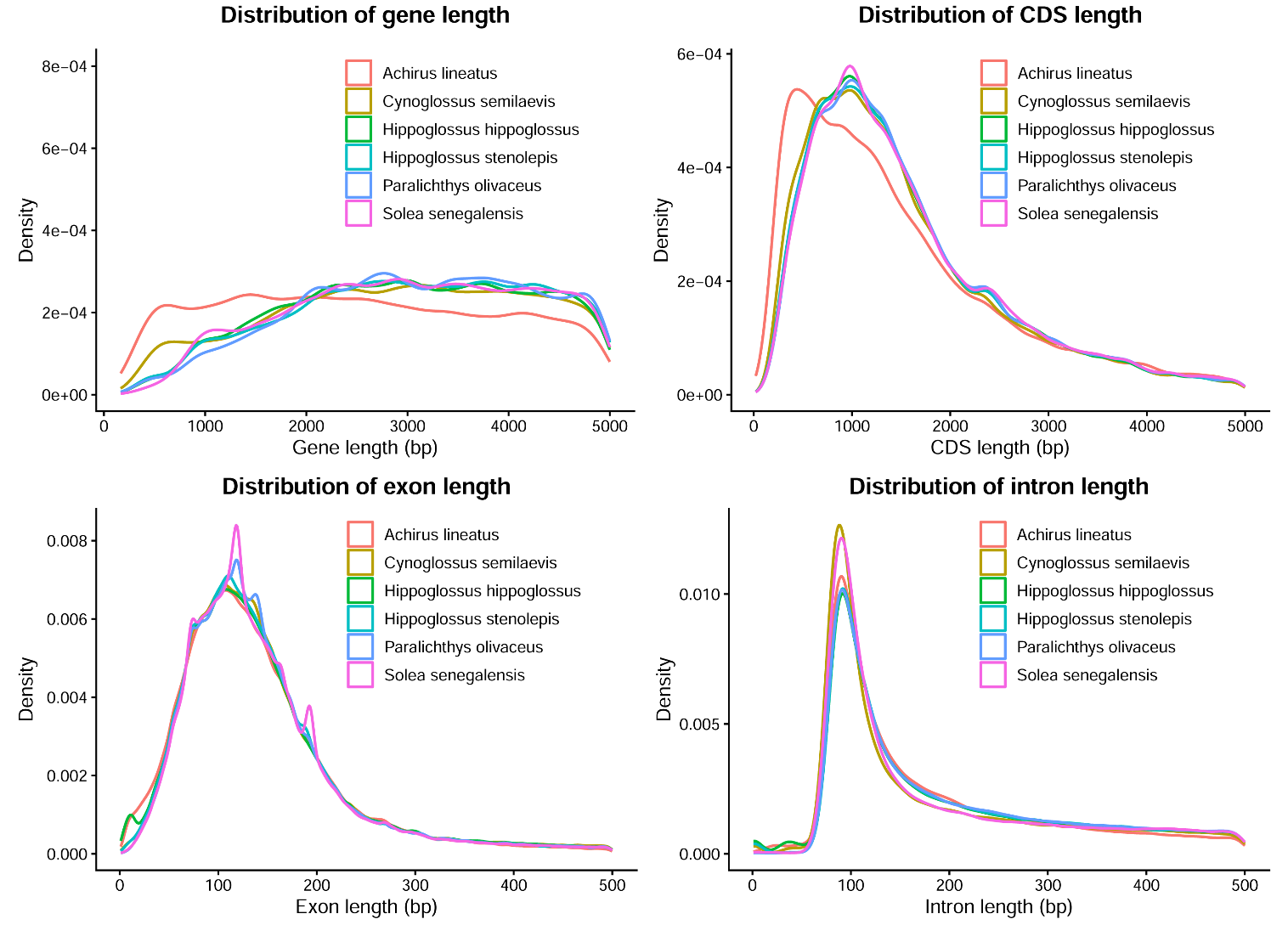


**Supplementary Figure 1** Comparative analysis of gene feature length distributions. The plots compare the length distributions of key gene structure between *Achirus lineatus* and other closely related species (*Paralichthys olivaceus, Cynoglossus semilaevis, Hippoglossus hippoglossus, Hippoglossus stenolepis* and *Solea solea*)*.* Top left: overall gene length; top right: coding sequence (CDS) length; bottom left: exon length; bottom right: intron length.


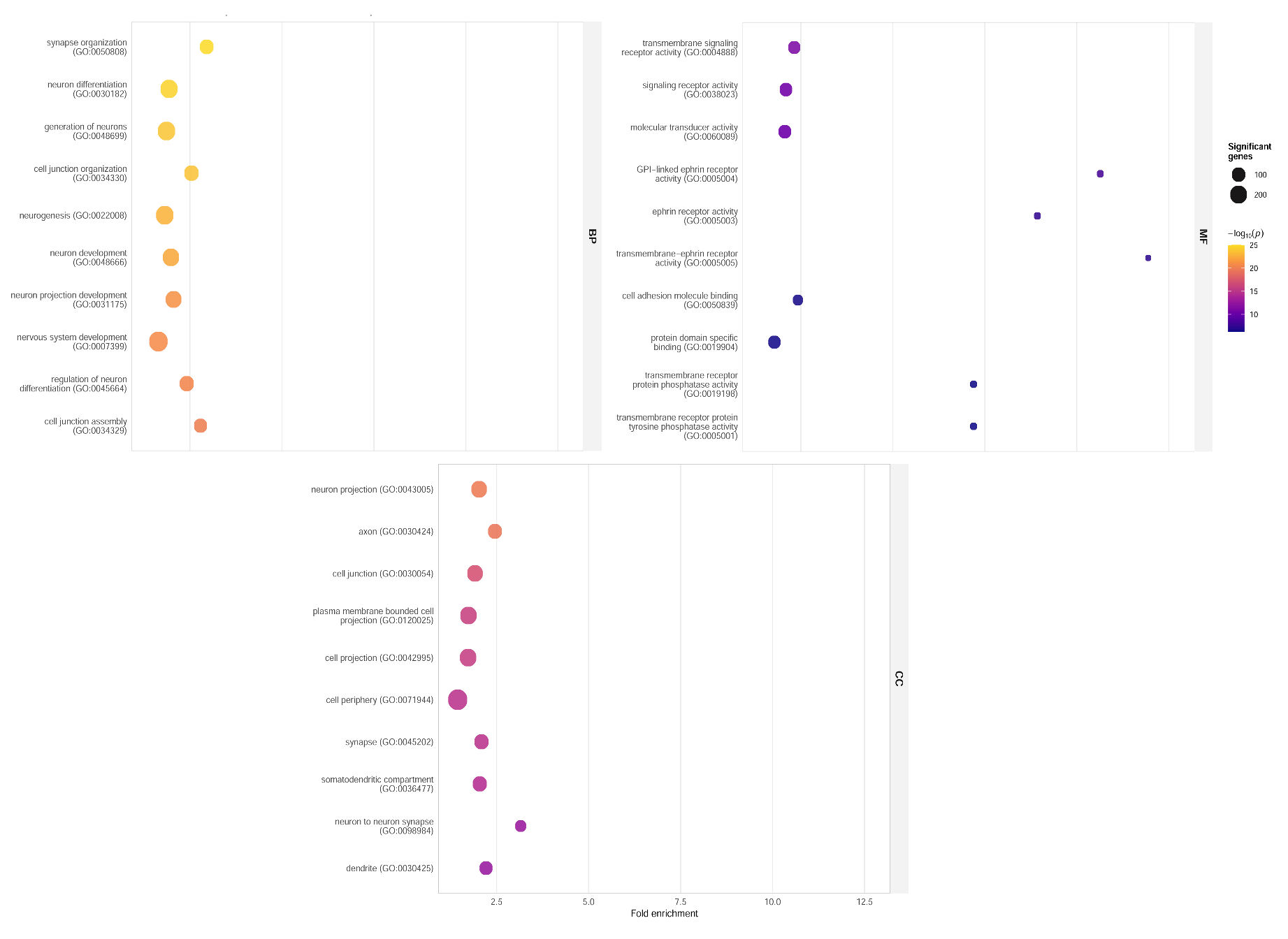


**Supplementary Figure 2** Top enriched GO terms in expanded orthologous families of *Achirus lineatus* (*p* < 0.05). BP=biological process, CC=cellular component, and MF=molecular function categories

**
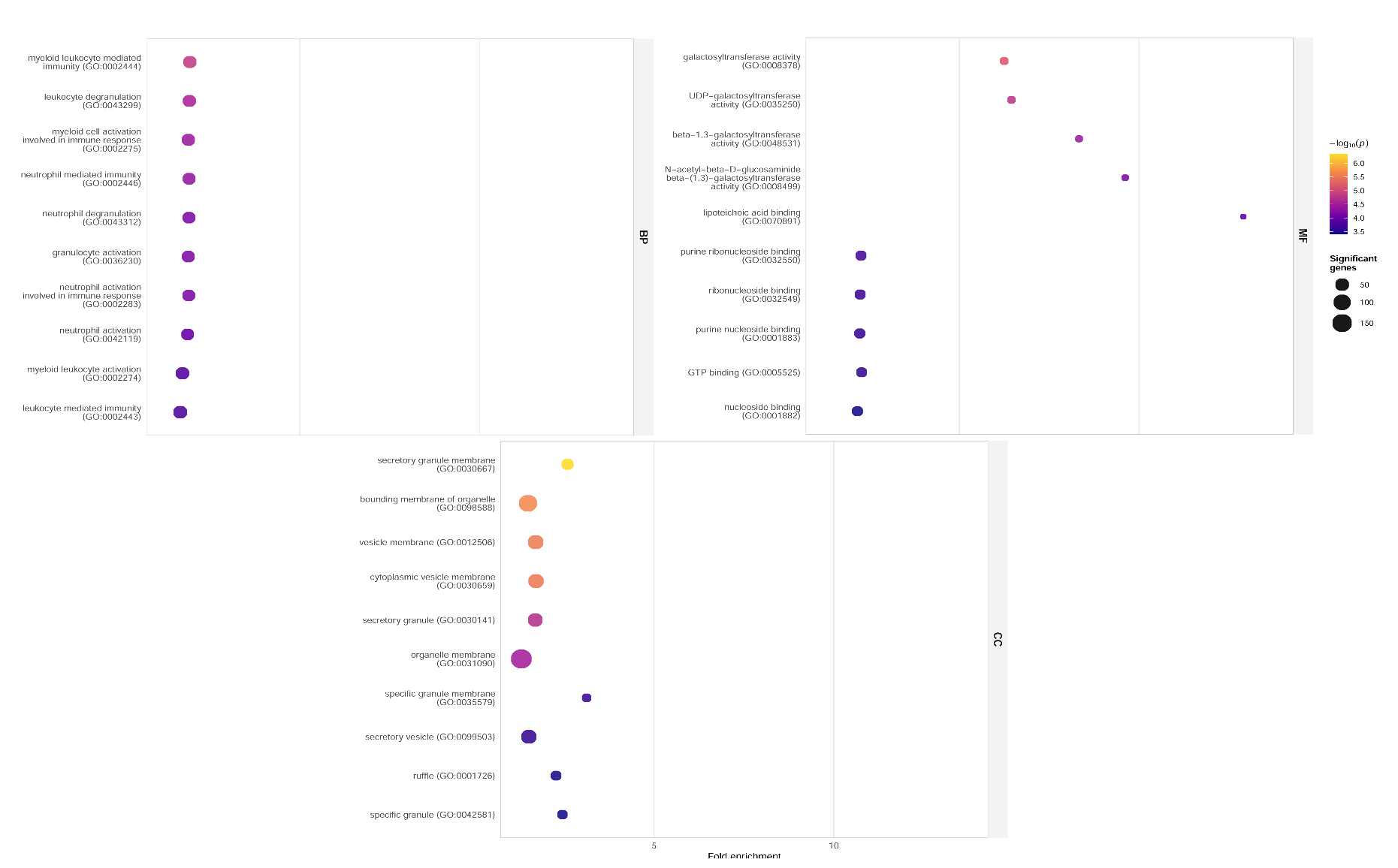
**

**Supplementary Figure 3** Top enriched GO terms in contracted orthologous families of *Achirus lineatus* (*p* < 0.05). BP=biological process, CC=cellular component, and MF=molecular function categories
